# Designing an evidence-based working method for medical work disability prognosis evaluation–an intervention mapping approach

**DOI:** 10.3389/fpubh.2023.1112683

**Published:** 2023-09-08

**Authors:** Sylvia P. Snoeck-Krygsman, Birgit H. P. M. Donker-Cools, Lyanne P. Jansen, Jan L. Hoving, Frederieke G. Schaafsma

**Affiliations:** ^1^Department of Public and Occupational Health, Amsterdam Public Health Research Institute, University of Amsterdam, Amsterdam, Netherlands; ^2^Research Center for Insurance Medicine (KCVG), Collaboration between AMC–UMCG–UWV–VUmc, Amsterdam, Netherlands; ^3^Department of Social Medical Affairs (SMZ), The Dutch Social Security Institute, The Institute for Employee Benefits Schemes (UWV), Amsterdam, Netherlands

**Keywords:** prognosis assessment, intervention mapping, functional abilities, evidence-based medicine, disability evaluation, insurance medicine

## Abstract

**Purpose:**

Performing evidence-based work disability prognosis evaluation (WDPE) of clients on sick leave is a difficult task for physicians. The aim was to develop a working method to support physicians in performing evidence-based WDPE and to improve WDPE quality.

**Materials and methods:**

Intervention Mapping (IM) supplemented with elements of the Behavior Change Wheel (BCW) guided project planning for developing the working method. This approach allowed combination with other frameworks and, e.g., behavior change theories. WDPE quality challenges were analyzed on various ecological levels, e.g., the individual (i.e., the physician), interpersonal (i.e., the client) and organizational level, culminating into a multilevel logic model of the problem. Determinants that contributed to this problem, e.g., lack of physicians’ knowledge on performing evidence-based WDPE, were identified. Performance objectives were formulated that could contribute to a desired change in WDPE quality. From the performance objectives and determinants (e.g., knowledge), change objectives were derived. In order to achieve these change objectives, suitable intervention functions (e.g., education) and policy categories (e.g., service provision) were identified, allowing the formulation of intervention components. Behavior change techniques (e.g., feedback on outcomes of a behavior) were selected to serve the intervention functions to deliver the desired change. This led to the conceptualization of an intervention plan.

**Results:**

The intervention “Prognosable” is presented. It consists of a stepwise working method (SWM) for evidence-based WDPE. The SWM offers an overview of important aspects (e.g., medical condition, clients’ confidence in return-to-work) to consider in individual clients’ WDPE. The SWM helps physicians to identify crucial functional limitations, find and appraise evidence-based information, weigh all relevant prognostic aspects and it supports physicians to conclude with an evidence-based WDPE, tailored to the individual client. The intervention “Prognosable” was designed, which also includes an educational program and a supportive software tool to enable implementation of the SWM.

**Conclusion:**

IM combined with BCW elements guided the development of a SWM for evidence-based WDPE. The SWM will be delivered through an educational program for physicians supported by a digital tool. The SWM, educational program and digital tool are ready to be implemented and evaluated in practice as the intervention “Prognosable.”

## 1. Introduction

Medical prognosis evaluation is considered a challenging task by various physicians (1, 2), in which errors are frequently made (3–5). Disability evaluations address functional abilities for work (6). Work disability prognosis evaluation (WDPE) therefore concerns the future perspective of these functional abilities. WDPE is one of the core tasks for insurance physicians (6) and many knowledge questions concern the topic of prognosis (7). For patients, WDPE is of great importance, as it involves perspectives on their future health, career and income. It should incorporate disease knowledge, predictive factors regarding sick leave, treatment and recovery behavior (8). However, WDPE is often not executed in such comprehensive manner (9). Insurance physicians performing WDPEs consider this task challenging, especially for cases with comorbidity or subjective health complaints (2).

The principles of evidence-based medicine (EBM) (10) are essential for WDPE quality (2). These EBM principles offer a method of clinical reasoning and decision making for the individual patient in which professional expertise is combined with the best available evidence (10). It involves formulating an answerable clinical question, searching for evidence, appraising the evidence, applying the evidence and the personal evaluation of the performance on a regular basis (11).

It was demonstrated that it was possible to practice EBM also in the areas of occupational (12) and insurance (13) medicine. Also, questions regarding disability prognosis could be answered by using the principles of EBM (2, 14, 15). In spite of advantages on quality and job satisfaction (16), actual EBM usage remains limited due to various barriers, especially the perceived time-investment (17).

Because of these challenges, insurance physicians have expressed a need for more support during WDPE (2, 17). To our knowledge, no WDPE working method exists. Several studies have now provided more insight into the content and process of WDPE [e.g., (17, 18)]. A variety of aspects was identified which insurance physicians consider in WDPE (17), such as the severity of the disease and the clients’ expectations of returning to work. Also, observations were made on the relative importance of different types of aspects (e.g., treatment aspects versus patient-related aspects) in different situations (e.g., degenerative conditions or rehabilitation purposes) (18). These studies also showed the various needs that physicians have, difficulties they experience and potential solutions they suggested to support them in WDPE. Suggestions included an overview of relevant WDPE aspects, help in the usage of EBM principles within insurance medicine and time-saving strategies, as confirmed in earlier studies (13, 19, 20).

The aim of this study is to develop a WDPE working method to support physicians in WDPE with the usage of EBM principles. It was expected that the consequent and adequate usage of a well-founded WDPE working method would increase EBM usage and improve WDPE quality. Increased EBM usage and improved substantiation will be beneficial for the transparency of the evaluation. This might also lead to a better acceptance of the outcome by clients. Because of the content as well as contextual complexity of WDPE, the Intervention Mapping (IM) protocol was used (21) to guide the project. It was believed that it would offer a systematic and comprehensive framework to assist in developing the intervention.

This paper describes the development of a WDPE working method intervention, using Intervention Mapping. Two design questions will be addressed:

1. What should be the content of a working method for disability prognosis evaluations?
2. How should such a working method be implemented?

## 2. Materials and methods

IM (21) was used as to guide the design of the working method and to plan its implementation and evaluation. IM provided a stepwise intervention planning framework. By means of problem and change modeling, ingredients for an intervention can be derived and design drafts can be made. For filling in the models, elements of the Behavior Change Wheel (22, 23) (BCW) were used supplementarily. The BCW offered nonredundant, comprehensive sets of determinants, intervention functions and behavior change techniques to select from.

The IM steps are shown in Supplementary Table S1, with permission of the author Kok G for the use of this figure (21). It entails six consecutive steps, but in an iterative way, to develop, implement and evaluate an intervention in a structured manner.

### 2.1. Step 1: logic model of the problem

Step 1 of the IM protocol (21) includes performing a needs assessment and an analysis of the intervention context, which then leads to the conceptualization of a logic model of the problem. The present study started with the analysis of the intervention context and the construction of a logic model of the problem, as a needs assessment was already performed in earlier studies (17, 18). A planning group was established, extended on various occasions with expert stakeholders.

Kox et al. (17) assessed content aspects, needs, barriers and solutions to consider in disability prognosis evaluations. They identified 23 aspects for consideration by insurance physicians (e.g., ‘nature of the disease’). Needs included education whereas barriers concerned time limits, difficulties with complex or vague diseases and the difficulty to apply evidence to an individual case. Suggested solutions included tools such as checklists or software applications with prognostic evidence (17). An additional study further evaluated the perceived importance of all these WDPE aspects (18). It showed that medical aspects (such as treatment) were valued most in practice, but that non-medical aspects might become more relevant in less clear conditions and that these could have important influence on rehabilitation outcomes. This knowledge was supplemented with outcomes from other studies [e.g., (2, 9, 14, 15, 17, 24–30)], mainly identified through cross-referencing. Also, outcomes of stakeholder meetings were added to these findings. This prior knowledge was used as input for problem modeling.

#### 2.1.1. Intervention context and program goals

An ecological levels approach (31), as suggested in the IM guide (21), was used to search for important, but accessible actors on various levels (e.g., on the individual level, on the organizational level, etc.). Characteristics and assets were identified which were present in their social, physical, informational and policy/practice environments (31). Next, program goals were defined.

#### 2.1.2. Logic model of the problem

A problem modeling template from the IM guide (21) was used, based on PRECEDE (32, 33), a health program planning model. Although the elements of this model concern public health problems (33), various earlier studies in the field of insurance medicine [e.g., (34–37)] demonstrated that problems could be modeled and addressed with IM. Therefore, “WDPE problems” was used as substitute for the construct “health problems” and “social security outcomes” was used instead of “quality of life.”

### 2.2. Step 2: logic model of change

With the ingredients of Step 2, a logic model of change was constructed.

#### 2.2.1. Behavioral and environmental outcomes

Within the planning group, it was discussed what would be the opposite (desired outcomes) of the behavioral and environmental outcomes from the logic model of the problem. These desired outcomes marked the endpoints for the logic model of change. Next, for every actor the detailed behavior [e.g., who, what, when, how often, with whom (23)] was described that should achieve the desired outcomes. Although the clients are important stakeholders and the recipients of WDPE, they were considered not to be actors that should be directly subjected to behavior change strategies within the intervention. Most of their performance objectives (e.g., “client understands what kind of outcome and consequences WDPE may yield”) were thought to be achievable through the IPs. Therefore, they were not shown as separate intervention target in the logic model of change.

#### 2.2.2. Performance objectives

The aforementioned description of the desired behavioral outcome was converted into one or more performance objectives (such as “the IP takes a structured, evidence-based approach in WDPE”) and split into their sequential steps (e.g., participating in education, using provided tools).

#### 2.2.3. Selecting determinants

As determinants, we used the 14 domains of the Theoretical Domains Framework (TDF) (38), because they are consistent with all known behavior change theory constructs (39) and include all key determinants form behavior theories (40–42). These domains (determinants) can be arranged under a uniform overarching model, COM-B (22), meaning that Behavior (B) is determined by the Capacity (C), Opportunity (O) and Motivation (M) for the individual or physician to perform it. The TDF-domains and their overarching COM-B components are illustrated in Supplementary Table S2. For every actor, each determinant’s contribution to WDPE problems as well as to WDPE change was worked out.

#### 2.2.4. Matrices of change objectives

For each actor, a matrix of change was made, by crossing the performance objectives with each of the TDF determinants, thus providing change objectives.

### 2.3. Step 3: program design

#### 2.3.1. The WDPE working method

The actual content of the WDPE working method was designed in parallel with the intervention program. We started out with the basic principles of EBM (10) and added information from stakeholder input, theories and evidence. These included, for example, the aspects worth considering in WDPE (17), content and design requirements [e.g., (2, 17, 43)] and outcome quality conditions and characteristics [e.g., (8, 16, 44)]. With these requirements and conditions and the findings from the formal IM steps, a concept WDPE working method was designed.

#### 2.3.2. Intervention mapping for program design

Program themes, components, scope and sequence were gradually specified in planning group meetings and stakeholder contacts. To select the most important determinants (e.g., ‘Knowledge’), it was explored for each of 14 TDF determinants whether a change would result in the desired behavior (23). For example, when the determinant “Knowledge” would change, physicians would know what quality and comprehensiveness is needed for high quality WDPE, how to substantiate their judgment with evidence and which approach to take. The behavior change objective “physician knows the ingredients of a good quality WDPE and is familiar with the way to obtain it” would then be met. The most relevant determinants, in terms of best meeting the change objectives, were selected.

The BCW offered a set of nine intervention functions (e.g., “Education” or “Restriction”) and provided the set of APEASE-criteria (Affordability, Practicability, Effectiveness, Acceptability, Side-effects/Safety and Equity). These can be used to determine which intervention function would best target the found determinants (22).

Next, the same criteria were used to identify which of the BCW’s set of seven policy categories (e.g., “Service provision” or “Guidelines”) would best carry out the intervention functions found (23). Then, behavior change techniques (45, 46) were sought which best targeted the determinants with the intervention functions (e.g., “Feedback provision” could be a technique to improve the determinant “Knowledge” and could be part of the intervention function “Education”). In order to provide a rationale for the problem and the intervention and to guide more detailed design choices, a suitable behavior change theory (45, 47) was searched for, which used the identified behavior change techniques as constructs (e.g., a well-founded theory that explains how “Feedback provision” could bring about a desired behavior change). A detailed scheme of the selection processes for determinants, intervention functions, policy categories, behavior change techniques and theory is depicted in Supplementary Table S3.

By applying the theory to each of the actors, practical applications were deduced. Then, intervention and its components were drafted. During design meetings with the planning group, it was checked whether the change objectives were still met by the various concepts of the intervention.

## 3. Results

### 3.1. Step 1: logic model of the problem

The planning group consisted of two behavioral scientists (psychologists) (SS-K, LJ), one occupational health physician (FS), two insurance physicians who routinely perform WDPEs (BD-C, SS-K) and one clinical epidemiologist (JH). Stakeholders from various expertise (e.g., managers, IT specialists, client representatives, medical staff, scientists, labor experts) collaborated or were consulted in differing frequencies throughout the project.

#### 3.1.1. Intervention context and program goals

By considering the ecological levels around the physicians, influencing the WDPE quality, four important actors to address were identified: Physicians (P) performing WDPEs were the main actor on the individual level. On the interpersonal level, the client (C), a claimant undergoing WDPE was an important actor. The organization (O), which contracts the physicians and provides disability benefits and vocational rehabilitation assistance to clients, was identified on the organizational level. And on the community level, the professional community of the physicians (PC) was found as a relevant actor. Regarding the societal level, there was not a specific actor isolated to direct the intervention to. The intervention was developed within the societal level of the existing social security system in the Netherlands. An elaboration on the needs and contexts of these actors is shown in Supplementary Table S4. From this elaboration, program goals were distilled. For example, to ensure transparency and uniformity on the one hand, and to involve unique client and context characteristics on the other hand, an important goal was the development of a stepwise, but client-tailored WDPE working method. The limited time but meanwhile high quality demands, led to the adoption of optimal efficient WDPE EBM support as a goal.

#### 3.1.2. Logic model of the problem

**Figure 1:**
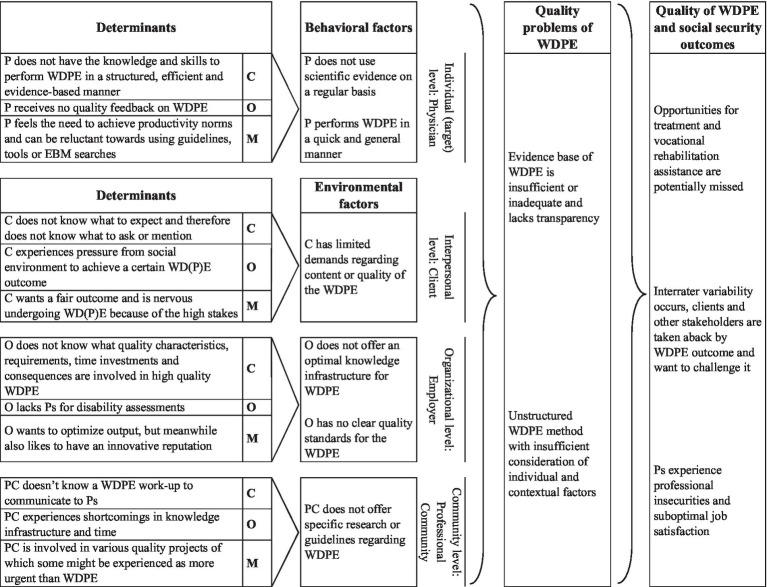
Logic Model of the Problem. P, Physician (an insurance physician performing disability evaluations); WDPE, Work Disability Prognosis Evaluation; C, Client (a disability claimant, patient); O, Organization (employer of physicians and provider of disability benefits); PC, Professional Community of the physicians (e.g., peers, colleagues); C (second column), Capacity (from COM-B model); O (second column), Opportunity (from COM-B model); M (second column), Motivation (from COM-B model).

The Logic Model of the Problem is presented in Figure 1. In the right panel, it shows that problems within WDPE could lead to missed chances for vocational rehabilitation, unsatisfied stakeholders and physicians experiencing professional insecurities. These can arise from an unstructured WDPE approach or low quality evidence base for the prognosis assessment. Behavioral and environmental factors from the various levels included, for example, a lack of feedback on the provided quality by the physician. The left part of the table shows the determinants underlying the behavioral and environmental factors.

### 3.2. Step 2: logic model of change

#### 3.2.1. Behavioral and environmental outcomes

An ideal outcome would be that WDPE consequences for benefit admission and assistance in vocational rehabilitation perfectly match the client’s health, contextual and personal factors, and that all actors are satisfied with the WDPE process and outcomes. Several requirements of WDPE quality and context can contribute to this outcome. One of these is a stronger evidence base, as this would increase quality and transparency (48). The setting in which WD(P) E is performed should offer an optimal knowledge infrastructure (49), to enable physicians to quickly identify and apply the latest scientific insights into their WDPEs. Also, a comprehensive set of personal or context related factors needs to be taken into account e.g., (17, 50, 51), to ensure optimal tailoring.

To achieve these outcomes, physicians must be regularly trained in EBM, and need to adhere to a stepwise protocol to guide them along the complex WDPE process. The organization needs to support the physicians with time, information, infrastructure, feedback and education. Their professional community needs to stimulate and assist them in knowledge exchange.

#### 3.2.2. Performance objectives

The identified performance objectives are shown in short, without all underlying sequential steps in Table 1. Because the intervention will not target the clients’ behavior directly, separate performance objectives and determinants for them are not listed in this table.

**Table 1:** Target behaviors and performance objectives for each actor.

Actor	Target behavior	Performance objectives
Physician (P)	The physician continually self-educates and trains to deliver high quality Work Disability Prognosis Evaluations (WDPE)s.	1. P takes a structured, evidence-based approach in WDPE. 2. P uses input from the client to personalize WDPE. 3. P promotes WD(P)E knowledge and development of herself and others in the profession.
Organization (O)	The organization puts effort in optimal knowledge infrastructure for physicians.	1. O facilitates the development, use and maintenance of a digital WDPE assistance tool. 2. O facilitates education and research on prognosis assessment. 3. O stimulates measurable quality standards in WDPE and ensures feedback on quality.
Professional Community of Physicians (PC)	The professional community stimulates WDPE knowledge exchange within and outside their community.	1. PC promotes the continuous professional development of Ps. 2. PC stimulates WD(P)E knowledge development. 3. PC exchanges knowledge on WDPE with other professional communities.

#### 3.2.3. Determinants and change objectives

The contribution of each of 14 TDF determinants on WDPE problems was worked out as well as what needs to be changed into for an ideal outcome. As an example, the TDF determinant *Memory, attention and decision processes* refers to the possibility that insurance physicians may lack an overview of relevant prognostic factors in WDPE as they are distracted by organizational demands and time pressure. It should change into physicians easily performing the WDPE steps, because they have acquired a routine, a certain speed and are familiar with its structure. This enables them to cope with and include other stakeholder demands. When crossing each of these determinants with each of the performance objectives, change objectives were obtained. As an example, the TDF determinant *Cognitive and interpersonal skills* within physicians, when crossed with the performance objective of taking a structured and evidence-based approach in WDPE, resulted in the change objective: “Physician is skilled in following the WDPE working method’s steps, in overviewing WDPE aspects, in using evidence, in weighing information and in communicating a well-substantiated outcome with the client and involved others.”

#### 3.2.4. Logic model of change

**Figure 2:**
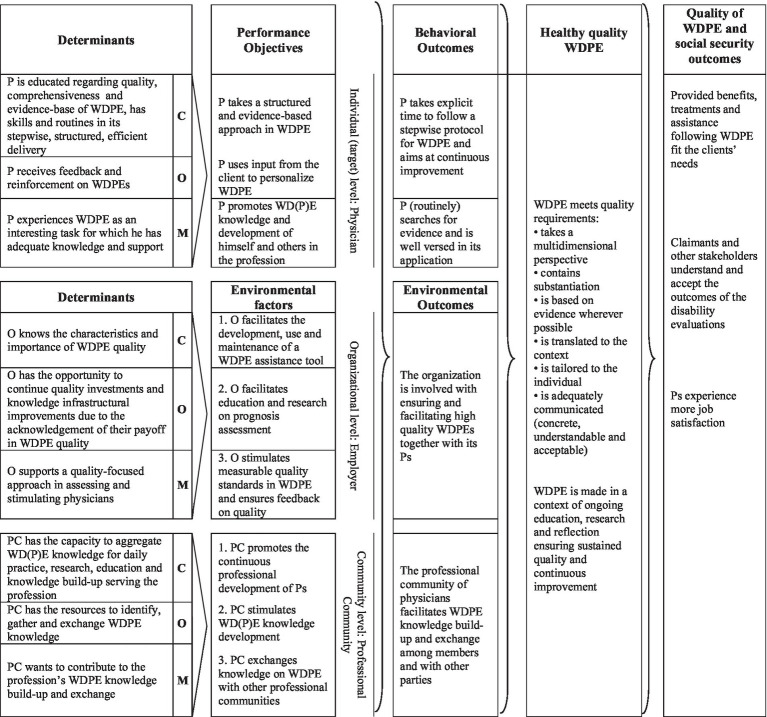
Logic Model of Change. The TDF-determinants are summarized into their overarching COM-B determinants. P, Physician (an insurance physician performing disability evaluations); WDPE, Work Disability Prognosis Evaluation; C, Client (a disability claimant, patient); O, Organization (employer of physicians and provider of disability benefits); PC, Professional Community of the physicians (e.g., peers, colleagues); C (second column), Capacity (from COM-B model); O (second column), Opportunity (from COM-B model); M (second column), Motivation (from COM-B model).

The logic model of change is shown in Figure 2.

### 3.3. Step 3: program design

#### 3.3.1. The WDPE working method

Based on all relevant aspects and needs for WDPE (17, 18), a five-step plan working method, accompanied by a training and a supporting tool was developed to meet the program goals. This working method, “Prognosable,” is shown in Supplementary Table S5. The overview as proposed in the “Prognosable” working method uses the International Classification of Functioning, Disability and Health (ICF) (52) in combination with the construct “Course” to also represent a time frame (26). For the construct of “Personal Factors” within the ICF, a set of ten main cognitions and perceptions influencing vocational rehabilitation previously identified by de Wit and colleagues, was used (53). To obtain a set of the most important aspects concerning “Environmental Factors” within the ICF, the ICF core sets of insurance and occupational medicine and the set from the Social Medicine Work Capacity instrument (51, 54–56) were used.

#### 3.3.2. Intervention mapping for program design

An overview of determinants, intervention functions and policy categories for each actor, which informed the WDPE method development, can be found in Supplementary Table S6. The importance of *Education* as an intervention function, combined with the policy category *Service provision*, led to the addition of an educational program to teach and train physicians in the WDPE method. The importance of *Enablement* as an intervention function, informed our choice to offer the WDPE method as a supporting software tool. Some derived practical applications regarding the educational program and the software tool are also briefly outlined in Supplementary Table S5.

##### 3.3.2.1. Physicians

Physicians are the main targets. It was aimed to improve the knowledge and the beliefs of the capabilities of the physicians by offering them services directed at education, enablement and training. The educational goals are related to consistently apply a structured stepwise evidence-based WDPE method in practice. In addition, the education and training will address personal development and growth of the profession by using feedback provision and encouraging knowledge exchange. The results indicated that adequate feedback provision would be an important behavior change technique for this target population. A relevant theory (57) was identified, offering an explanation for findings in earlier studies that physicians who were educated in the use of guidelines or other evidence sources, still found it hard to consistently use these in practice [e.g., (16, 20, 58)]. For example, when physicians take efforts to deliver high quality evidence based disability evaluations, but do not receive any positive feedback from their supervisor or the organization, internal mechanisms will influence their motivation and decrease their intentions to take these efforts again. Thus, our intervention should include positive forms of feedback toward the physicians reinforcing WDPE quality efforts, promoting this otherwise hard-to-maintain behavior. As a practical application, *direct experience* (21, 59), could be a way to provide feedback. When a physician has repeated *performance mastery experiences* either in training or in actual WDPE practice, his self-efficacy and attempts to achieve such results again will increase (59). Test questions in the training combined with practice outcome experience might enforce this behavior and contribute to internalization.

##### 3.3.2.2. Organization

For the organization, ‘Beliefs about consequences’ also was an important determinant, next to ‘Intentions’. The organization lacks physicians and that could lead to valuing quantitative over qualitative outputs. This might reduce the perception of the benefits of good quality WDPE for its tasks and mission. To improve the knowledge and perception of qualitative output consequences and to stimulate WDPE quality initiatives, educative and persuasive communications can be used. Environmental and social planning (such as an intranet page or internal news messages on the intervention) might achieve this by broadcasting good WDPE quality results or promising WDPE pilots. This way, a behavioral incentive is offered for striving toward high quality WDPE. Another environmental modification can be the incorporation of WDPE quality as a standard topic in quality meetings and performance evaluations. This serves the purpose of feedback provision toward physicians as well as of improving the organization’s beliefs about the consequences of WDPE quality. Therefore, these strategies could accompany the delivery of the WDPE tool and training.

##### 3.3.2.3. Professional community of physicians

Although the focus of the intervention is on the individual physician, their professional community of peers and other colleagues was also studied. For the individual physician, knowledge and skills will be improved by the educational program. For the physicians, as a group, a more emotional determinant was found, which can be addressed by increasing professional confidence, pride and ambitions. Here, communication and marketing (for example on case examples, examples of good WDPE practice, useful information sources) can be of assistance. This might encourage reflection and contribute to personal development and growth of the profession, which is consistent with the fifth step of the EBM method (11).

### 3.4. Preparing steps 4 to 6: production, implementation, evaluation

The program production will involve the development of a software tool and a training program to support and educate the “Prognosable” working method. Drafts and demos will first be shown to expert physicians for refinement. Then, a limited efficacy testing study with case vignette illustrations will be performed among physicians to obtain information on the feasibility of the working method’s steps and conceptualization. Also, the feasibility of the training program for its education and the software tool as means of support will be tested. A final version of the “Prognosable” working method, training program and software tool will then be made and tested on its effect on quality and its implementation in actual practice.

## 4. Discussion

### 4.1. Main findings

With the help of IM and elements of the BCW, a new comprehensive working method for disability prognosis evaluations was conceptualized. It offers a structured approach with an overview of aspects, in which professional freedom is obtained to describe and relate these aspects to the prognosis of functioning for each unique client.

It is summarized in a combined intervention consisting of a training program and a digital support tool. The training and the tool will guide the physician through the stepwise working method, based on EBM principles and addressing specific prognostic considerations for the insurance medicine context.

### 4.2. Comparison with the literature

#### 4.2.1. Using program planning frameworks

Since 2007, various studies in occupational and insurance medicine have used IM for intervention development [e.g., (35–37, 60, 61)] and some have used the BCW (e.g. (62, 63),). Some of the studies using IM, reported advantages for intervention development such as meeting the specific needs of the actors and targeting specific behavioral determinants (e.g., “Knowledge”). However, a systematic review by Fassier et al. (34) on studies using IM for developing return-to-work interventions, demonstrated that the effectiveness of the implemented interventions was often limited. They reported that possible causes included a not truly participative planning group, a somewhat randomly chosen behavior change theory and not involving workplace actors in intervention development (34). In our study we therefore chose to include targeted actors in the planning group and to hold stakeholder meetings with others. Also, we chose to use a behavior change theory based on required constructs (covering the identified determinants and behavior change techniques) and specific insights (see Supplementary Table S3). Moreover, because we chose a multilevel approach with the ‘organization’ as an actor in itself, this workplace actor was integrated in the development of the intervention.

#### 4.2.2. Prognosis evaluation assistance

Several tools and algorithms exist to calculate prognostic outcomes or to suggest possible treatment interventions for certain prognostic risk factors. Examples in the field of disability evaluations include Louwerse et al. (29, 64), who developed a prediction rule to predict future changes in work ability. De Wit et al. (53, 65) presented an overview of interventions to alter negative cognitions and perceptions for return-to-work. In addition, Hesse et al. (27, 66) developed a list of indicators, which assist in determining acceptance for long-term disability benefits for people with psychiatric illnesses. None of these, however, described a complete working method, a generic work-up for disability prognosis evaluation, such as was developed in this study.

Because difficulties in prognosis assessment included early steps (such as gathering information) as well as late steps (such as a concrete and well-substantiated reporting of the evaluation), the entire prognosis evaluation process was captured within the working method. However, earlier studies on guidelines and prediction rules indicated that physicians tend to fear a loss of professional autonomy when these are too strict, whereas when they are too generic, they are not considered helpful (28). Therefore, the intervention with a stepwise working method should offer guidance and overview, while preserving all individual case-specific characteristics and professional freedom within the steps.

#### 4.2.3. Education

As knowledge was an important determinant, education was an important intervention function. Several other studies in the field of occupational or insurance medicine pursued physicians’ behavior change, for example toward guideline adherence. They all identified needs in knowledge and skills (29, 30, 37, 62), often leading to an educational program as an appropriate, acceptable and feasible intervention component. Moreover, stakeholders in our study mentioned that the only well-implemented interventions targeting physicians within this organization, were delivered through education. In addition, this education would need to be part of the mandatory training program for residents and highly recommended for the registered insurance physicians.

In this study, feedback provision appeared to be a very important behavior change technique, which was included into the educational program by means of exercises. For practicality and cost-effectiveness, part of the program can be offered by means of e-learning. De Leeuw and colleagues (67) described an evidence-and theory-based development strategy for digital medical education, which will be consulted. Its principles such as feedback provision, interactive elements, real-world translation, showing progress, reference provision and clear layout very well matched physicians’ needs and implementation strategies identified in this study. For the EBM education, a practice-integrated teaching method, as Coomarasamy and Khan (68) showed, would have beneficial outcomes on knowledge, skills, attitudes and behavior, whereas a standalone method might only improve knowledge. This will be realized by using realistic or actual cases and by using the working method and tool in the workplace setting.

#### 4.2.4. Tool

The working method will be supported by a software tool, which guides the physicians through all WDPE steps. For literature resources, immediately accessible hyperlinks to preselected evidence will be offered, as well as search strategies for additional resources. An underlying database consists of linking tables for diagnostic codes and their search terms with reference sources and for prognostic aspects and reference sources. This will help meeting physicians’ needs in evidence identification and might overcome time barriers (2, 20).

Also, an overview of aspects will be offered, as physicians suggested this kind of support (17, 18), indicating a need for information structuring. A study involving a prediction rule (43) showed that easy graphical representations were preferred design choices, which we will adhere to in the software development.

Although physicians need to make evidence-based considerations [e.g., (49, 69)] and although they received EBM training (13, 19), EBM usage remains limited [e.g., (16, 58)]. They still consider EBM usage difficult (20, 58) and are in need of EBM assistance (2, 17, 18). Moreover, since most EBM questions involved the topic of prognosis (7), EBM steps (such as finding, appraising and applying evidence) were integrally included within the working method and the education. For practice, a digital tool will be provided, with help in acquiring, searching and appraising evidence, such as direct links to relevant selected EBM resources, filters, search strings and appraisal questions.

### 4.3. Strengths and limitations

A strength of this study is that it combined IM with elements of the BCW. This might have captured the advantage of IM in reducing the chances of failures in conceptualization, implementation and theory application (34) and captured the advantage of the BCW for its structured, practical and transparent approach (70).

A limitation of this approach could be that it involved some interpretation and adjustments to smoothly combine them. For example, the BCW’s *behavior change techniques* (BCTs) were somewhat different from IM’s *theoretical methods*. The limited set of BCTs was used to choose from and IM’s theoretical methods were consulted, when more examples or detail was needed on supposedly similar concepts.

A systematic review on interventions targeted at health care professionals demonstrated that it was not uncommon to use parts of different frameworks together (71). Our study also complied to their identified four standard elements to consider (regardless of the framework (s) used) when developing an intervention involving knowledge translation: identification of barriers, selection of intervention components, using theories and involving end-users (71).

An advantage of the undertaken systematic work-up, is that implementation challenges (such as time constraints and educational needs) were identified in an early stage (the logic model of the problem). The comprehensive problem analysis led to an extensive description of the desired behavior changes. All of these needs and challenges ultimately led to design choices for implementation. The considerations made within the APEASE exercises offered guidance. For example, the need for education, feedback and help on the one hand versus the costs and productivity loss on the other hand, ended up in a short training, which included WDPE feedback exchange with peers, combined with support from the software tool in performing the working method’s steps. Moreover, because of the distinctly identified and described constructs, measures can be formulated through which future implementation can be readily assessed and adapted.

## 5. Conclusion

In this study, IM and components of the BCW were used to develop the disability prognosis evaluation working method ‘Prognosable’, a stepwise approach based on EBM principles. For its implementation, drafts for a training program and a supportive tool were also designed. A feasibility study with limited efficacy testing, will guide its further development. Next, with a multicenter single blinded randomized controlled trial and a subsequent practice evaluation, its quality and its implementation, respectively, will be evaluated and optimized.

## Data availability statement

The datasets presented in this article are not readily available because the datasets generated during and/or analyzed during the current study are available from the corresponding author on reasonable request. Requests to access the datasets should be directed to s.p.snoeck-krygsman@amsterdamumc.nl.

## Ethics statement

Ethical review and approval was not required for the study on human participants in accordance with the local legislation and institutional requirements. Written informed consent for participation was not required for this study in accordance with the national legislation and the institutional requirements.

## Author contributions

SS-K, BD-C, LJ, JH, and FS contributed to the study conception and design. SK wrote the first draft of the manuscript. All authors contributed to the article and approved the submitted version.

## Funding

The project was financed by the Dutch Institute of Employee Benefit Schemes (UWV), Amsterdam, The Netherlands, as part of the Research Center for Insurance Medicine (KCVG). However, no funding bodies had any role in study design, data collection and analysis, decision to publish, or preparation of the manuscript.

## Conflict of interest

The authors declare that the research was conducted in the absence of any commercial or financial relationships that could be construed as a potential conflict of interest.

## Publisher’s note

All claims expressed in this article are solely those of the authors and do not necessarily represent those of their affiliated organizations, or those of the publisher, the editors and the reviewers. Any product that may be evaluated in this article, or claim that may be made by its manufacturer, is not guaranteed or endorsed by the publisher.

## Figures and Tables

**Table:** 

APEASE	Affordability, Practicality, Effectivity, Acceptability, Side-effects/Safety, Equality
BCT	Behavior Change Technique
BCTTv1	Behavior Change Technique Taxonomy version 1
BCW	Behavior Change Wheel
C(s)	Client(s) claiming disability benefits and undergoing WDPE
CEOS-theory	Context, Executive and Operational Systems Theory
COM-B	Capacity, Opportunity and Motivation causing Behavior
WDPE	Work Disability Prognosis Evaluation
EBM	Evidence-Based Medicine
ICF	International Classification of Functioning, Disability and Health
IM	Intervention Mapping
O(s)	Organization (or its managers) contracting the physicians and offering or rejecting the disability benefits to the clients
P(s)	Physician(s) performing disability evaluations, here mainly social insurance physician(s)
PC(s)	Professional community (or its members) of the physicians
PO(s)	Performance Objective(s)
PICOTS	Patient, Intervention, Control, Outcome, Timeframe, Setting
PRECEDE	Predisposing, Reinforcing and Enabling Constructs in Educational Diagnosis and Evaluation
PROCEED	Policy, Regulatory, and Organizational Constructs in Educational and Environmental Development
TDF	Theoretical Domains Framework
WMA	World Medical Association
