# Supplementary material for: Designing an evidence-based working method for medical work disability prognosis evaluation–an intervention mapping approach
Source: Front Public Health. 2023 Sep 8;11:1112683. doi: 10.3389/fpubh.2023.1112683 (PMC10516134; doi:10.3389/fpubh.2023.1112683)
Supplement: Supplementary file 1 [file Table_1.pdf]

Designing an evidence-based working method for medical disability prognosis evaluation – an intervention mapping approach

Additional file 1: Intervention mapping steps

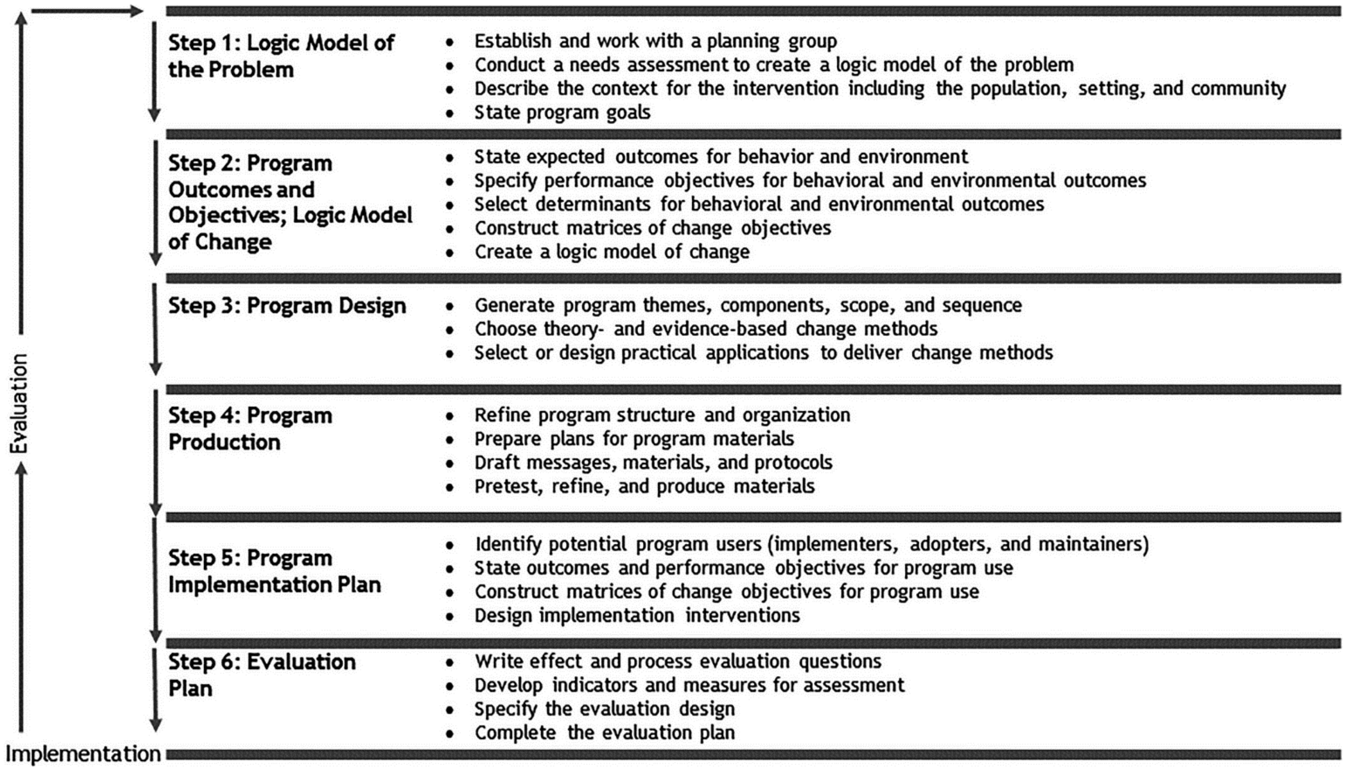

The six steps of Intervention Mapping [1], with permission of the author (G. J. Kok) for the use of this figure.

1. Bartholomew Eldredge LK, Markham CM, Ruiter RA, Fernández ME, Kok G, Parcel GS: **Planning health promotion programs: an intervention mapping approach**: John Wiley & Sons; 2016.
