# Supplementary material for: Designing an evidence-based working method for medical work disability prognosis evaluation–an intervention mapping approach
Source: Front Public Health. 2023 Sep 8;11:1112683. doi: 10.3389/fpubh.2023.1112683 (PMC10516134; doi:10.3389/fpubh.2023.1112683)
Supplement: Supplementary file 2 [file Table_2.pdf]

Designing an evidence-based working method for medical disability prognosis evaluation – an intervention mapping approach

Additional file 2: Domains of the Theoretical Domains Framework (TDF) and their contribution to the components of the COM-B model (Capacity-Opportunity-Motivation→Behavior) [1-4]

TDF-domains (determinants)[i]	COM-B components	
Knowledge	Psychological	**Capacity**
Cognitive and interpersonal skills		
Memory, Attention and Decision Processes		
Behavioral regulation		
Physical skills	Physical	
Social Influences	Social	**Opportunity**
Environmental context and resources	Physical	
Reinforcement	Automatic	**Motivation**
Emotion		
Social/Professional Role and Identity	Reflective	
Beliefs about Capabilities		
Optimism		
Intentions		
Goals		
Beliefs about Consequences		

References

1. Michie S, Johnston M, Abraham C, Lawton R, Parker D, Walker A, Psychological Theory G: **Making psychological theory useful for implementing evidence based practice: a consensus approach**. *Qual Saf Health Care* 2005, **14**(1):26-33.
2. Cane J, O'Connor D, Michie S: **Validation of the theoretical domains framework for use in behaviour change and implementation research**. *Implementation science* 2012, **7**(1):37.
3. Michie S, Van Stralen MM, West R: **The behaviour change wheel: a new method for characterising and designing behaviour change interventions**. *Implementation Science* 2011, **6**(1):42.
4. Michie S, Atkins L, West R, Goosen H, van't Hof K, Mehra S: **Het gedragsveranderingswiel: 8 stappen naar succesvolle interventies**. Amsterdam: Amsterdam University Press; 2018.

[i] In this table 15 domains are shown, instead of 14. The determinant *Skills* is sometimes subdivided into *Cognitive and interpersonal skills* and *Physical skills*.
