# Supplementary material for: Designing an evidence-based working method for medical work disability prognosis evaluation–an intervention mapping approach
Source: Front Public Health. 2023 Sep 8;11:1112683. doi: 10.3389/fpubh.2023.1112683 (PMC10516134; doi:10.3389/fpubh.2023.1112683)
Supplement: Supplementary file 3 [file Table_3.pdf]

Designing an evidence-based working method for medical disability prognosis evaluation – an intervention mapping approach

Additional file 3: Study flow from determinants to theory selection

1. Determinant selection

Actor 1 (Physicians)

Performance objective 1: P takes a structured and evidence-based approach in DPE
Performance objective 2: P explicitly asks for and discusses clients' opinion during DPE
Performance objective 3: P follows courses, performs practice exercises and reflects on DPEs introspectively as well as with others

	Changed determinant		Change objective	Determinant change required? [1]	
Determinant 1: Knowledge	P knows what quality and comprehensiveness is needed, how to base this on evidence and knows a stepwise/structured approach to ensure these	for PO1:	P knows the ingredients of a good quality DPE and is familiar with the way to obtain it.	Y	**Determinant selected because majority of POs is addressed by its change.**
		for PO2:	P knows how to verbally and written communicate D(P)E (e.g. principles of expectation management, structuring arguments, motivational interviewing)	Y	
		for PO3:	P knows D(P)E quality characteristics and how to reflect on his work.	N (partially met by former CO and existing meetings)	

[Same procedure for determinant 2 to 14.]

[Same procedure for the other 3 actors.]

2. Intervention function identification

Actor 1 (Physicians)

Chosen determinants:	Possible intervention functions for the determinant[2]	A	P	E	A	S	E [3]	+		
Determinant 1: Knowledge	Education	y	y	y	y	y	y	6	An optimum between online and live lessons needs to be determined. E-learning modules will be less expensive and easier to offer in practice. However, live training might be more effective and better accepted. Equity can be supported by modular training, supporting different baseline knowledge levels.	**Intervention function selected (with APEASE-score of 6)**
Determinant 2: Cognitive and interpersonal skills	Training	?	?	y	y	?	?	2	This type of training will require a live setting with more suitable instructors. It might be perceived as personal/confronting. Due to differences in received attention in the training and differing quality of instructors, inequalities might arise.	Intervention function not selected for step 3 (because of APEASE-score < 4)
Determinant 4: Behavioral regulation	Education	y	n	?	y	y	?	0	Education for this determinant is not practical, as these skills require practice exercises.	Intervention function rejected, because of an APEASE-item with score 'no'.
	Training	[Same procedure for the other intervention functions]								
	Enablement									

[Same procedure for other selected determinants]

---

[1] As suggested in worksheets within the Behavior Change Wheel manual.
[2] A Matrix is offered within the Behavior Change Wheel manual in which possible intervention functions for each TDF-determinant are listed.
[3] The APEASE-criteria (Affordability, Practicality, Effectiveness, Acceptability, Side-effects / Safety and Equity) are offered within the Behavior Change Wheel manual for this selection.

[Same procedure for the other 3 actors.]

3. Policy category identification

Actor 1 (Physicians)

Selected intervention functions:	Possible policy categories for the intervention function [4]	A	P	E	A	S	E	+		
Intervention function 1: Education	Communication / Marketing	n						0	Financially not in proportion to other ways to achieve the same or better results on education.	Intervention function rejected, because of an APEASE-item with score 'no'.
	Service Provision	y	y	y	y	y	y	6	Providing services to offer education is a salient, affordable and acceptable policy category to address shortages in knowledge and educational needs derived from other determinants.	**Intervention function preselected (with APEASE-score of 6)**

[Same procedure for other selected intervention functions]

[Same procedure for the other 3 actors.]

4. Best pairs selection

Actor 1 (Physicians)

Determinant	Intervention function	Comment	APEASE Det-Int	Policy category	APEASE Int-Pol	Comment	Sum	
Knowledge	Education	Education by means of E-learning will be cheaper and more practical. However, live encounters might be more effective or better appreciated. Equity (considering end terms) might be most efficiently realized by providing learning modules dependent on the initial knowledge level. In this way, the somewhat tailored education, will improve the acceptance of E-learning.	6	Service provision	6	Providing services to offer education is a salient, affordable and acceptable policy category to address shortages in knowledge and educational needs derived from other determinants.	12	**Intervention function, policy category and determinant selected, because they belong to the two sets with the highest scores for the actor.**
Behavioral regulation	Training	Education has proven to be effective in changing physicians' practice behavior [1]. Some studies suggest that a training embedded within the actual practice might be preferrable [2]. It is however somewhat more complex to realize and some of the 'softer skills' (e.g. communication skills) might not be as readily accepted for training as others (e.g. searching skills). Use of practice enablers, such as flow charts, might have an extra effect on practice behavior [3] and was also suggested by our stakeholders.	4	Service provision	5	Services can realize training can be attended, within a course as well as 'on the job'. Some training effects can be provided by feedback provision and scheduling the topic (e.g. to demonstrate/discuss good practice), but also explicit time provision will be needed to practice and improve DPE skills.	9	Intervention function with policy category rejected, because it doesn't belong to the two most promising sets for the actor.

[4] A Matrix is offered within the Behavior Change Wheel manual in which possible policy categories for each intervention function are listed.

Beliefs about capabilities	Modeling	A training could demonstrate examples of good practice, offer inspiration and a frame of reference one is willing to adhere to and potentially increase professional confidence and pride.	6	Service provision	5	Within an educational service, elements of modeling can be of assistance to make the target behavior more clear (e.g. following the methodology, seeing what words and style to use in DPE communication, etc.) and easier to imitate.	11	**Intervention function, policy category and determinant selected, because they belong to the two sets with the highest scores for the actor.**

[Same procedure for other selected intervention functions and their selected policy categories]

[Same procedure for the other 3 actors.]

5. Intervention component formulation

… physicians: educational service to improve knowledge and modeling examples to improve belief in the capacities
… clients: communication and marketing (information) to improve their belief about the consequences with legislative information for persuasion
… organization: communication and marketing (broadcasting) as incentive for DPE quality supportive initiatives and environmental / social planning to restructure the environment in order to offer these quality rewards
… professional community of physicians: communication and marketing (information) to demonstrate and guide good practice and improve feelings of professional confidence

6. Behavior change technique identification

Actor 1 (Physicians)

Determinants selected in step 1 and intervention functions from step 2

Behavior change techniques: [6] [4, 5]	knowledge	memory, attention and decision processes [5]	behavioral regulation	environmental context and [illegible]	beliefs about capabilities	education	training	environmental and social restructuring	modeling	enablement	**sum**	
<u>2 Feedback and monitoring</u>												
2.1 Monitoring of behavior by others without feedback											0	Behavior change technique rejected, because it's not well suited for the determinants nor the intervention functions.
2.2 Feedback on behavior	**x**					**x**	**x**				3	**Behavior change technique listed as potentially useful for this actor, because of a sum score of at least 3.**
2.3 Self-monitoring of behavior			**x**			**x**	**x**			**x**	4	**Behavior change technique listed as potentially useful for this actor, because of a sum score of at least 3.**

[Same procedure for other of 93 behavior change techniques]

[Same procedure for the other 3 actors.]

7. Theory identification

Actor 1 (Physicians)

Behavior change techniques with scores >= 3.			Group
**score 4**	2.3	self-monitoring of behavior	2. Feedback and monitoring

---

[5] For this determinant, no behavior change techniques were listed.
[6] The behavior change techniques are described in the Behavior Change Technique Taxonomy version 1 (BCCTv1). In the Behavior Change Wheel guide, matrices are provided listing potential behavior change techniques for TDF-determinants and for intervention functions.

**score 3**	2.2	feedback on behavior	2. Feedback and monitoring
	2.4	self-monitoring of outcome(s) of behavior	2. Feedback and monitoring
	2.6	biofeedback	2. Feedback and monitoring
	4.4	behavioral experiments	4. Shaping knowledge
	7.1	prompts/cues	7. Associations
	7.2	cue signaling reward	7. Associations
	12.1	restructuring the physical environment	12. Antecedents
	12.2	restructuring the social environment	12. Antecedents

Group selected as main source of behavior change techniques for this actor.
Keywords "feedback" and "monitoring".
Searched for behavior change theories[7] [6] using "feedback*" and/or "monitor*" as constructs.

4. Behavioural-Ecological Model of Adolescent Aids Prevention (Hovell et al.)........ 63	N
**5. CEOS Theory (Borland).. 71**	Y
6. Change Theory (Lewin)... 79	N

[Same procedure for the other 80 theories of behavior change]

Actor 2 (Clients)

**score 4**	NA		
**score 3**	5.3	information about social and environmental consequences	5. Natural consequences
	5.6	information about emotional consequences	5. Natural consequences

Group selected as main source of behavior change techniques for this actor.
Keywords "consequences" or "outcome".
Searched for behavior change theories using "consequen*" or "outcome*" as constructs.

4. Behavioural-Ecological Model of Adolescent Aids Prevention (Hovell et al.)........ 63	Y
**5. CEOS Theory (Borland).. 71**	Y
6. Change Theory (Lewin)... 79	N

[Same procedure for the other 80 theories of behavior change]

[Same procedure for the other 2 actors.]

8. Theory selection

Preferred theories (covering all selected groups of behavior change techniques):	Year:		
5. CEOS Theory (Borland).. 71	2014	**Theories considered, because dated after insights of 2001 [8] [7, 8].**	**Preferred theory due to recency, involvement of rational and emotional mechanisms [9, 10] (as both were relevant for our actors), comprehensiveness, usability (detailed) and conceptualization of hard-to-maintain behavior changes.**
34. Integrated Theory of Health Behaviour Change (Ryan) 207	2009		Less preferred theory.
60. Social Action Theory (Ewart) 343	1991	Theories discarded, because dated before insights of 2001 [7, 8].	
77. Theory of Interpersonal Behaviour (Triandis) 423	1977		
80. Theory of Triadic Influence (Flay & Petraitis).. 437	1994		

[7] Theories were identified from the ABC of behavior change theories by searching for the keyword stems within the entire text.
[8] In 2001 a set of key determinants of behavior was discovered and attempts were made at constructing universal integrated behavior change theories.

This part of the study flow was largely based on working methods and exercises described in the Behavior Change Wheel manual [5]. The Behavior Change Technique Taxonomy [4] was used to identify behavior change techniques. The ABC of Behavior Change Theories [6] was used to find suitable theories of behavior change. The attempts from 2001 onwards to find universal behavior change theories [7, 8], were referred to as a reason to only consider the theories of more recent dates. Studies on motivation in the context of addiction showed the importance of considering a combination of rational as well as emotional mechanisms on behavior change [9, 10].

References

1. Forsetlund L, O'Brien MA, Forsen L, Reinar LM, Okwen MP, Horsley T, Rose CJ: **Continuing education meetings and workshops: effects on professional practice and healthcare outcomes**. *Cochrane Database Syst Rev* 2021, **9**:CD003030.
2. Kok R, Hoving JL, Smits PB, Ketelaar SM, van Dijk FJ, Verbeek JH: **A clinically integrated post-graduate training programme in evidence-based medicine versus ‘no intervention’for improving disability evaluations: a cluster randomised clinical trial**. *PLoS One* 2013, **8**(3):e57256.
3. Davis D, Davis N: **Selecting educational interventions for knowledge translation**. *CMAJ* 2010, **182**(2):E89-93.
4. Michie S, Richardson M, Johnston M, Abraham C, Francis J, Hardeman W, Eccles MP, Cane J, Wood CE: **The behavior change technique taxonomy (v1) of 93 hierarchically clustered techniques: building an international consensus for the reporting of behavior change interventions**. *Ann Behav Med* 2013, **46**(1):81-95.
5. Michie S, Atkins L, West R, Goosen H, van't Hof K, Mehra S: **Het gedragsveranderingswiel: 8 stappen naar succesvolle interventies**. Amsterdam: Amsterdam University Press; 2018.
6. Michie S, West R, Campbell R, Brown J, Gainforth H: **ABC of Behaviour Change Theories: An Essential Resource for Researchers, Policy Makers and Practitioners**: Silverback Publishing; 2014.
7. Fishbein M, Triandis HC, Kanfer FH, Becker M, Middlestadt SE, Eichler A: **Factors influencing behavior and behavior change**. *Handbook of health psychology* 2001(1):3-17.
8. Montaño D, Kaspryzk D: **Theory of reasoned action, theory of planned behavior, and the integrated behavior model**. In: *Glanz, K, Rimer, BK, & Viswanath, K (2008) Health Behavior and Health Education: Theory, Research, and Practice.* edn.; 2008: 67-92.
9. **Prime Theory of Motivation - Theory of Motivation** [www.primetheory.com]
10. West R, Brown J: **Theory of addiction**: John Wiley & Sons; 2013.
