# Supplementary material for: Designing an evidence-based working method for medical work disability prognosis evaluation–an intervention mapping approach
Source: Front Public Health. 2023 Sep 8;11:1112683. doi: 10.3389/fpubh.2023.1112683 (PMC10516134; doi:10.3389/fpubh.2023.1112683)
Supplement: Supplementary file 4 [file Table_4.pdf]

Designing an evidence-based working method for medical disability prognosis evaluation – an intervention mapping approach

Additional file 4: Needs and contexts of the four actors

Physicians

The physical environment of physicians while performing DPE is often a doctor's office. [i] This office can be located within a governmental building or a health clinic, but does not usually have hospital facilities. [i] Therefore, extended examinations or imaging cannot be carried out and there's no laboratory equipment. [i] Content information for the assessment is obtained from medical records, history taking and basic examination of the client, contact with others (e.g. treating physician) and internet searches for evidence. [i]

There is no methodology for DPE yet. [i] Physicians know [i] that it should concern the functional abilities (e.g. [1]) and that EBM-usage is strongly recommended for quality and transparency (e.g. [2]). According to physicians in occupational health, the organization does not provide adequate access to knowledge sources and they experience an inadequate knowledge infrastructure [3-5]. Although IT infrastructure and access to knowledge sources have improved profoundly, this presented new problems: how to identify the best evidence within an overload of information and how to translate and apply this in actual practice, in an efficient way (e.g. [3]). Work-related outcomes (such as functional abilities) don't have clear, uniform definitions [6]. The development of tools such as search filters [7-9] and ICF core sets [6, 10] could facilitate DPE evidence identification. For example, search filters could assist in identifying studies on disability and or on prognosis. ICF core sets could help to describe functioning and relevant aspects in a more uniform way. That could lead to studies using more uniform concepts and variables, which can be retrieved easier by physicians.

EBM courses could assist with identification and application, but although various EBM courses have offered, many physicians still not often consult evidence sources [7, 11, 12] and factors such as reluctance [5], lack of time [3, 4, 12, 13] and application problems [13] still seem to play a role. Due to this infrequent EBM practice, the acquisition of habitual EBM skills is jeopardized. With the complex task of DPE and high time pressure, physicians will try whether a short general DPE will suffice [14, 15] and risk their argumentation becoming feeble [16]: scarce arguments with weak foundation. Especially how improvement could be achieved [15] or why it can't be achieved [14], is often not too well documented. Guidelines and tools could facilitate EBM usage in practice [13]. However, studies on guideline adherence and propositions of ready-made evidence-based prediction rules, met skepticism: physicians were unfamiliar with them, mistrusted their results and sometimes feared a loss of professional freedom in decision-making and room to weigh the claimants wishes [17, 18].

One of the social/cultural contributions to DPE problems is that physicians are trained to be doctors. [i] They have taken an oath on not inflicting harm on patients. [i] Some physicians are afraid that prognosis communication can result in adverse effects [13, 19, 20], such as losing hope or motivation. High demands are placed on DPE quality. [i] DPE often implies not only a survival prediction, but also a forecast of increasing or decreasing handicaps and their eventual consequences for work functioning. [i] Descriptions of quality demands included for example testability, reproducibility and consistency, preferably achieved by a foundation in scientific evidence [21, 22].

Due to a lack of physicians, there are also considerable demands on productivity. [i] This contributes to physicians feeling not being adequately supported (provided with time and help for this complex task) and experience professional insecurities pertaining DPE [13]. Conversely, a study on training and support led to increased D(P)E quality and job satisfaction [2].

Various physicians mentioned that they rarely received feedback on quality. [i, ii] The lack of guidance on how to perform DPE in general, along with the time pressure, can contribute to an unstructured DPE method, in which not all aspects of consideration are taken into account.

Clients

Clients come from very different backgrounds, with very different health problems and very different societal contexts and demands. [iii] A DPE assessment often involves a one-time encounter with the physician. [i] Clients tend to be very nervous for this assessment and there can be very high stakes (e.g. keeping their job, receiving enough income, having perspectives of societal participation). [i]

Shortcomings in DPE could result in interventions and disability benefits not matching with the health status or capacities of the client [23, 24]. This might lead to them performing inadequate recovery behavior and thereby missing opportunities for recovery and rehabilitation [13].

Clients do not fully oversee the consequences of DPE [i, iii]. Therefore the client does not know what to ask for. Also, they can be overwhelmed by emotions, the load of information and the importance of D(P)E for his future. [i] His adequate contribution to DPE might be jeopardized by these forces. [i, ii]

They have the right to receive a fair treatment [i, iii, iv]. When inter- and intra-physician differences are noticed, which potentially could lead to differences in outcomes [20, 25], clients might question the fairness of their D(P)E. They even might feel disadvantaged and dissatisfied, which can cause reputational damage [26] to physicians, the organization and their professional community. On the other hand, client stakeholders mentioned that "one size fits all" doesn't apply here. [iii] They do want to be evaluated with careful consideration of their unique characteristics. [iii]

Organization

The organization can be a governmental or corporate institution. [i] Its main client is society as a whole and its task is to provide social security for working-aged citizens. [i] Financing is received from insurance premiums by employers and employees and by contributions from the ministry. [i] Employers and employees can claim various kinds of disability coverage. [i]

Society demands a timely and high quality output from the organization.[v] Because the organization experiences a shortage of physicians with D(P)E expertise[, i, ii, iv, v] it is difficult to meet these requirements. Board and staff stakeholders mentioned that they sometimes feel reluctant towards the education of physicians, as this decreases their output on the short term, because they need to invest their time in the training. [iv]

Governmental institutions receive a lot of media attention. [i, v] Clients, their employers and society members in general all have different and often conflicting interests. [i] The organization's output is critically evaluated by them and sometimes this is broadcasted in the media. [i, ii, v] Its professional reputation is very important for society's trust in their government and in their social security. [i, v] This makes the organization destined to make the low number of available physicians perform as much D(P)Es as possible, but also of the highest quality. A tailored approach was met with enthusiasm by stakeholders from the organization, given that it would not take (significantly) more time. [iv] In contrast, managers also mentioned that they liked to participate in pilot studies and new ideas. [iv, v] This could contribute to future efficiency and could also be beneficial for their reputation as being innovative. [i, iv, v]

Its managers, however, are often not content experts on DPEs. [i, ii, v] They can provide feedback and management on the quantitative outputs of physicians, but might be less equipped regarding the qualitative outputs. This can cause relatively more vigorous actions directed at quantity compared to quality. Physicians might feel being left alone with a difficult task, without any content help and with a high time pressure. [i, ii]

Professional community of the insurance physicians

Although the professional community of physicians developed various guidelines, these often concern specific diseases. [i] And research mostly addresses interventions for vocational rehabilitation and the prognosis of return-to-work instead of the prognosis of the functional abilities themselves. [i, ii]

To our knowledge there's no general guideline or method that specifically concerns DPE.

In order to assess the disability prognosis, a structured method assessing functional abilities is considered most transparent and empirically sound [27]. However, to date it remains unknown how

to obtain a standardized measure of 'work capacity', best reflecting functional limitations and their interaction with work environments [27]. Prognosis of disabilities for social insurance purposes has not been studied extensively and not much guidance exists for professional decision making [28]. Many physicians developed their own style. [i, ii] Their opinions on the importance of different areas of aspects for DPE seems to differ considerably [i]. Differing opinions also exist towards the exact definition of *Functional Capacities* [29] and how these should be measured and quantified. There's a need for uniformity and guidelines on this topic, which has been recognized by various national and international communities (e.g. [10]).
The field of medical disability evaluations is relatively novel and research tradition is therefore not as extensive as in some clinical specialties. [i] This, combined with the high societal demands, has made its professional community eager to increase its scientific knowledge base to be acknowledged in its expertise. [i, ii, v] Because of the significant differences in national disability regulations, international comparability of study outcomes can be very difficult (e.g. [29]). This might put a constraint on the speed of growth of the scientific knowledge base, [i] compared to other medical fields and can give rise to professional insecurities. [i, ii]

Needs and contexts of the four actors: physicians, clients, the organization and the professional community of the physicians.

These descriptions are made by combining evidence from theory, from empirical studies and from stakeholder meetings. Also, we have drawn conclusions based on this information. Information from theory and evidence sources is shown as reference. Information from stakeholders is indicated by footnotes. The remainder stems from our modeling and mapping of this information and the conclusions we drew from it.

Literature references:

1. de Boer W: **Quality of evaluation of work disability**. *Academisch proefschrift Universiteit van Amsterdam Hoofddorp* 2010.
2. Hoving JL, Kok R, Ketelaar SM, Smits PB, van Dijk FJ, Verbeek JH: **Improved quality and more attractive work by applying EBM in disability evaluations: A qualitative survey**. *BMC Medical Education* 2016, **16**(1):77.
3. van Dijk FJ, Verbeek JH, Hoving JL, Hulshof CT: **A knowledge infrastructure for occupational safety and health**. *Journal of occupational and environmental medicine* 2010, **52**(12):1262-1268.
4. Hoving JL, van der Voort R, Kok R, Verbeek JH, Hulshof CT: **Het belang van een onderbouwde prognose en de rol van evidence daarbij: een survey onder verzekeringsartsen**. *TBV–Tijdschrift voor Bedrijfs-en Verzekeringsgeneeskunde* 2016, **24**(10):465-471.
5. Schaafsma F, Hulshof C, Van Dijk F, Verbeek J: **Information demands of occupational health physicians and their attitude towards evidence-based medicine**. *Scandinavian journal of work, environment & health* 2004:327-330.
6. Brage S, Donceel P, Falez F: **Development of ICF core set for disability evaluation in social security**. *Disability and rehabilitation* 2008, **30**(18):1392-1396.
7. Geersing G-J, Bouwmeester W, Zuithoff P, Spijker R, Leeflang M, Moons K: **Search filters for finding prognostic and diagnostic prediction studies in Medline to enhance systematic reviews**. *PloS one* 2012, **7**(2):e32844.
8. Haafkens J, Moerman C, Schuring M, van Dijk F: **Searching bibliographic databases for literature on chronic disease and work participation**. *Occupational medicine* 2005, **56**(1):39-45.
9. Kok R, Verbeek JA, Faber B, van Dijk FJ, Hoving JL: **A search strategy to identify studies on the prognosis of work disability: A diagnostic test framework**. *BMJ open* 2015, **5**(5):e006315.
10. Anner J, Brage S, Donceel P, Falez F, Freudenstein R, Oancea C, de Boer W: **Validation of the EUMASS Core Set for medical evaluation of work disability**. *Disability and rehabilitation* 2013, **35**(25):2147-2156.
11. Verhaaf M, Hoving J: **Evidence-based verzekeringsgeneeskunde**. *TBV–Tijdschrift voor Bedrijfs-en Verzekeringsgeneeskunde* 2018, **26**(2):59-66.
12. Kok R, Hoving JL, Verbeek J, Schaafsma FG, van Dijk FJ: **Integrating evidence in disability evaluation by social insurance physicians**. *Scandinavian journal of work, environment & health* 2011:494-501.
13. Kox RJ, Hoving JL, Verbeek JH, Schouten MJ, Hulshof CT, Wind H, Frings-Dresen MH: **Assessment of prognosis by physicians involved in work disability evaluation: A qualitative study**. *PloS one* 2019, **14**(2):e0212276.
14. Ankersmit JF, Chan WK, Kok R, Wind H, Hoving J: **Onderbouwing van de duurzaamheid bij Wajong 2015 beoordelingen**. *TBV–Tijdschrift voor Bedrijfs-en Verzekeringsgeneeskunde* 2018, **26**(10):518-525.
15. Hesse B: **Entwicklung einer Indikatorenliste zur sozialmedizinischen Beurtei-lung der Reintegrationsprognose und der Rehabilitationsbedürftigkeit bei Rentenantragstellern mit psychischen Erkrankungen (IREPRO)**. *des NRW-Forschungsverbundes Rehabilitationswissenschaften* 2006:134-146.
16. Gezondheidsraad: **Verzekeringsgeneeskundige mediprudentie**. In.: Gezondheidsraad (GR) (Health Council of the Netherlands); 2007: 1-58.

17. van Muijen P, Duijts SF, Kornet-van der Aa DA, van der Beek AJ, Anema JR: **Work disability assessment of cancer survivors: insurance physicians' perspectives**. *Occup Med (Lond)* 2015, **65**(7):558-563.
18. Louwerse I, Huysmans M, van Rijssen H, Gielen C, van der Beek A, Anema J: **Use of a Decision Support Tool on Prognosis of Work Ability in Work Disability Assessments: An Experimental Study Among Insurance Physicians**. *Journal of Occupational Rehabilitation* 2020:1-12.
19. An HJ, Jeon HJ, Chun SH, Jung HA, Ahn HK, Lee KH, Kim M-h, Kim JH, Cheon J, Kim J: **Feasibility study of physician orders for life-sustaining treatment for patients with terminal cancer**. *Cancer Research and Treatment: official journal of Korean Cancer Association* 2019, **51**(4):1632.
20. Muller E, Hoving J: **Visie van verzekeringsartsen op de claimbeoordeling van mensen met een beperkte levensverwachting**. *TBV–Tijdschrift voor Bedrijfs-en Verzekeringsgeneeskunde* 2018, **26**(10):533-537.
21. Gezondheidsraad: **Verzekeringsgeneeskundige protocollen: Algemene inleiding, Overspanning, Depressieve stoornis**. In*.* The Hague; 2006.
22. Raad voor Gezondheidsonderzoek (RGO): **Advies onderzoek verzekeringsgeneeskunde**. In*.* The Hague: Raad voor Gezondheidsonderzoek (RGO): 1-60.
23. Wijnia JW, Corstiaensen IJ: **A poor prognosis: guide or misleading?** *American Journal of Hospice and Palliative Medicine®* 2008, **25**(1):5-8.
24. Stattin M: **Retirement on grounds of ill health**. *Occupational and Environmental Medicine* 2005, **62**(2):135-140.
25. Rios Garcia M, Spanjer J: **De invloed van de diagnose op de beoordeling van belastbaarheid**. *Tijdschrift voor Bedrijfs- en Verzekeringsgeneeskunde* 2020, **28**(4):20-25.
26. Marfeo EE, Haley SM, Jette AM, Eisen SV, Ni P, Bogusz K, Meterko M, McDonough CM, Chan L, Brandt DE: **Conceptual foundation for measures of physical function and behavioral health function for social security work disability evaluation**. *Archives of physical medicine and rehabilitation* 2013, **94**(9):1645-1652. e1642.
27. Baumberg Geiger B, Garthwaite K, Warren J, Bambra C: **Assessing work disability for social security benefits: international models for the direct assessment of work capacity**. *Disability and rehabilitation* 2018, **40**(24):2962-2970.
28. Hesse B, Gebauer E, Heuft G: **Die IREPRO-Indikatorenliste-eine Arbeitshilfe zur systematischen Beurteilung von Reintegrations-Prognose und Rehabilitationsbedürftigkeit in der psychiatrischen Rentenbegutachtung**. *Die Rehabilitation* 2007, **46**(01):24-32.
29. Anner J, Kunz R, Boer Wd: **Reporting about disability evaluation in European countries**. *Disability and Rehabilitation* 2014, **36**(10):848-854.

Stakeholder information:

[i] Knowledge or experience of stakeholders within our planning group.
[ii] Encounters with stakeholder physicians in meetings unrelated to this study.
[iii] Meeting with client representatives about this study.
[iv] Meeting with organization representatives about this study.
[v] Encounters with stakeholders from the organization (e.g. managers, staff, board members), unrelated to this study.
