# Supplementary material for: Designing an evidence-based working method for medical work disability prognosis evaluation–an intervention mapping approach
Source: Front Public Health. 2023 Sep 8;11:1112683. doi: 10.3389/fpubh.2023.1112683 (PMC10516134; doi:10.3389/fpubh.2023.1112683)
Supplement: Supplementary file 5 [file Table_5.pdf]

Designing an evidence-based working method for medical disability prognosis evaluation – an intervention mapping approach

Additional file 5: Prognosable

PROGNOSABLE				
**Methodology**			**Learning service**	**Tool service**
**Step**	**Activity**	**Illustration**		
0	It is assumed that the physician starts the methodology steps when the social-medical history, the interventions and the functional capacities of the client are already investigated.	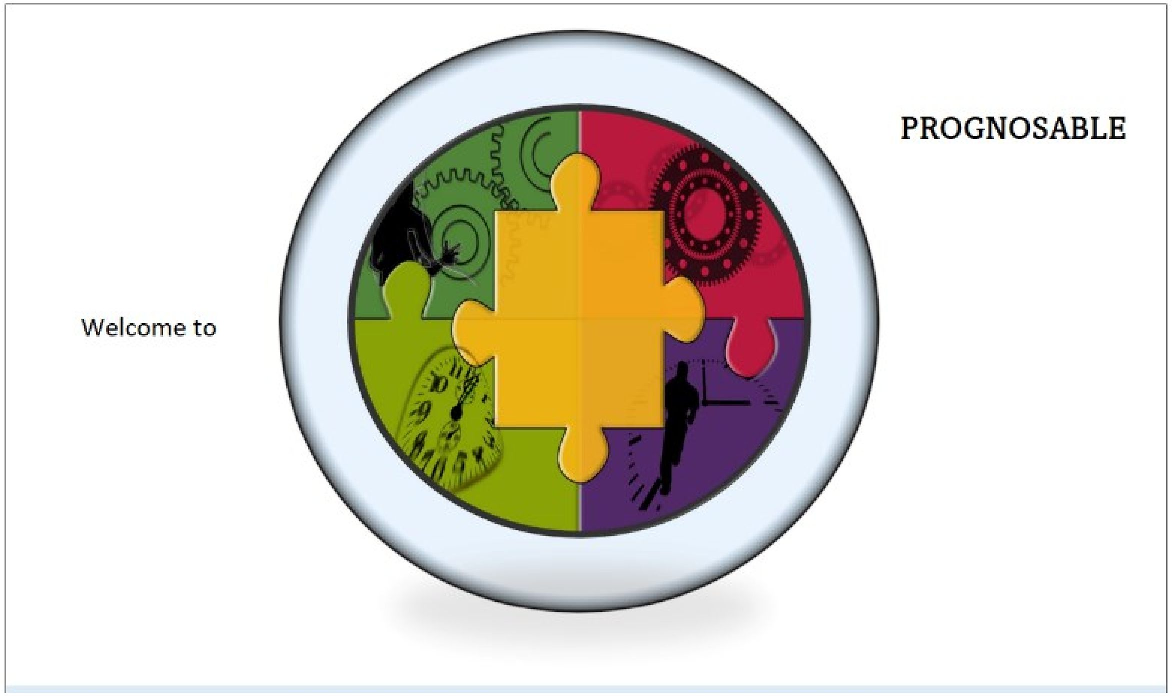	Not applicable	Not applicable

1	The physician collects information on various aspects of influence on the prognosis of the client's functioning. The aspects are grouped within domains around the construct of functioning, based on the ICF framework representation.	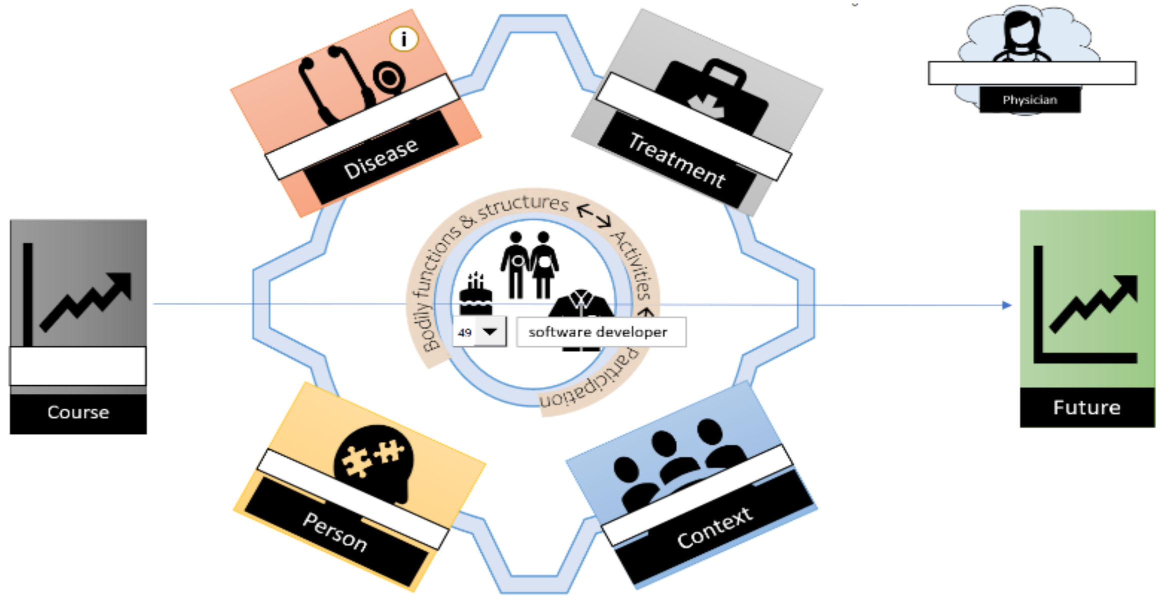	The physician learns about the domains and important aspects and prognostic factors. The conceptualization within the modified ICF-framework is taught and practical exercises are offered (e.g. think of an important prognostic aspect from each domain for this case vignette).	In the first two steps, the tool offers overview and assists in structuring information with list boxes and text note boxes.

<table>
<tr>
<td>2</td>
<td>The physician identifies ‘bottle-neck’ disabilities, formulates a prognostic question about their potential improvement, finds out what information is needed to answer it and rephrases the question accordingly into PICOTS terms.</td>
<td>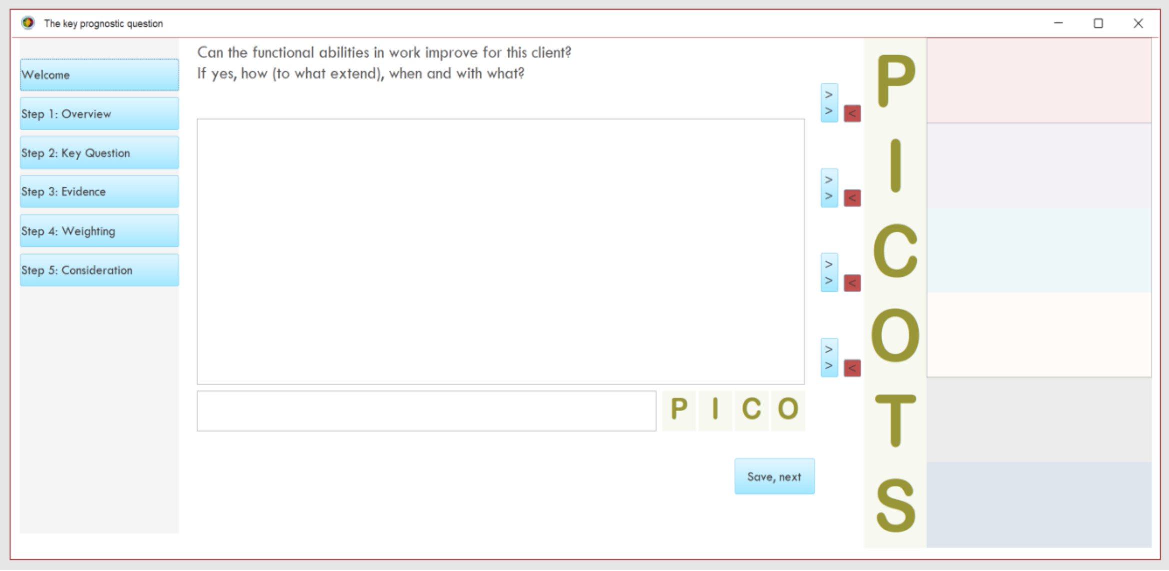
</td>
<td>Practical exercises are offered to help identify the disabilities with most impact on work functioning. Also, there will be exercises on formulating a key prognostic question, identifying information needs and on extracting PICOTS terms.</td>
<td></td>
</tr>
</table>

<table>
<tr>
<td>3</td>
<td>The physician searches, selects, appraises and applies the evidence.</td>
<td>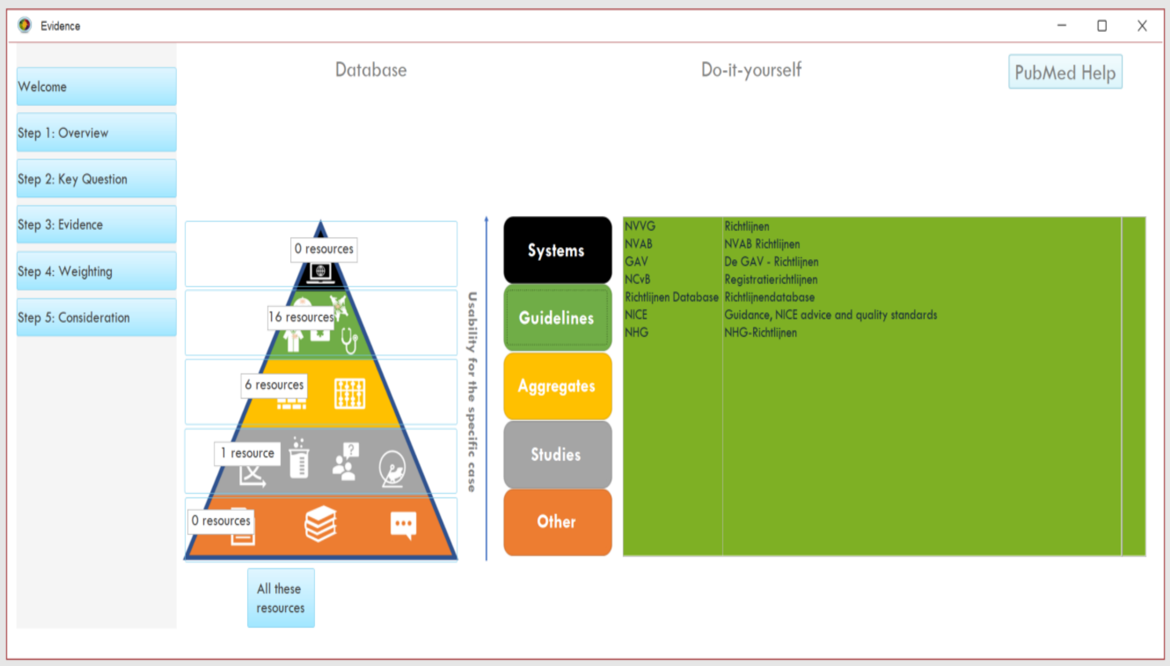
</td>
<td>This is taught by exercises on evidence sources, selecting relevant information, substantiating the relevance and critical appraisal. Thus, these steps mainly teach and train the concept of EBM. Since EBM experience might vary profoundly between physicians, these steps might be offered in different variants: e.g. short for EBM experts, intermediate as 'refresher course' and extended for novices.</td>
<td>Links to relevant sources are offered and guidance is offered through the EBM steps. And it offers speed by automation. For example, search strings are generated based on selected diagnosis and desired filters.</td>
</tr>
</table>

<table>
<tr>
<td>4</td>
<td>The physician weighs all the information on all aspects.</td>
<td>
<table>
<tr><td>Weighting of relevant aspects:</td><td></td><td>Contribution:</td><td>Substantiation:</td><td>Sources:</td></tr>
<tr><td>Condition:</td><td>Category IV degeneration</td><td>x</td><td></td><td></td></tr>
<tr><td>Treatment:</td><td>Awaits knee-replacement surgery.</td><td>+</td><td>will improve walking, stair-climbing</td><td>1</td></tr>
<tr><td>Course:</td><td>Surgery got postponed frequently.</td><td>+</td><td></td><td></td></tr>
<tr><td>Patient factors:</td><td>Is afraid that surgery might fail.</td><td>-</td><td>can benefit from psychological intervention</td><td>4</td></tr>
<tr><td>Context factors:</td><td>Runs his own business. Can't find employees to do the physical demanding tasks.</td><td>0</td><td></td><td></td></tr>
<tr><td>(Physician-related considerations)</td><td>0</td><td>x</td><td></td><td></td></tr>
</table>
</td>
<td>Exercises are offered with case vignettes to assign values to information on aspects based on source, quality, relevance, etc.</td>
<td>Links to tools and instruction videos are provided. Also, important prognostic factors are shown along with particular selected diseases and/or aspects.</td>
</tr>
<tr>
<td>5</td>
<td>The physician formulates a well-substantiated answer on the prognostic key question and pays attention to stakeholder demands and needs in the final formulation.</td>
<td>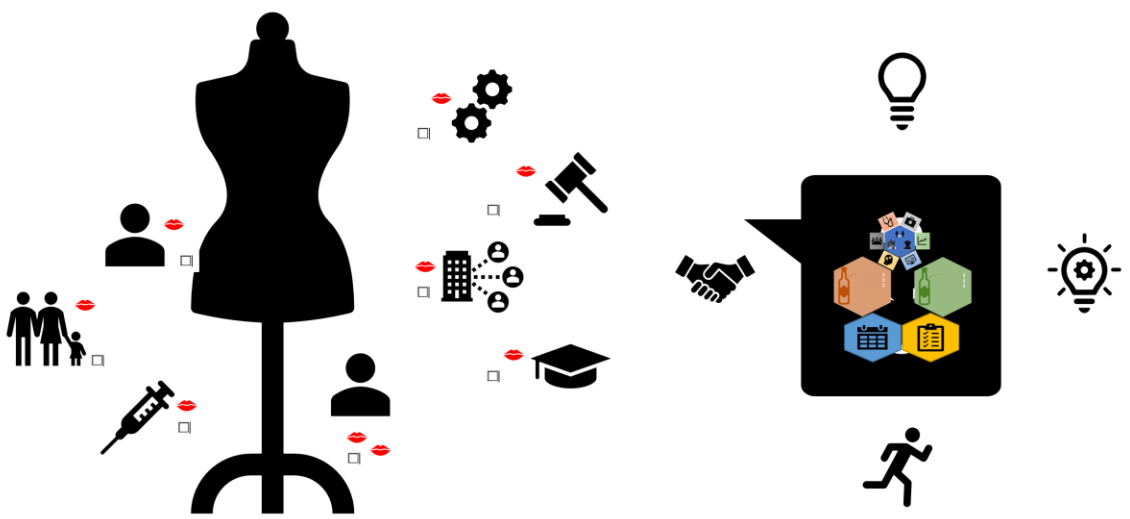</td>
<td>Actual cases will be used to practice the argumentative prognostic reasoning and substantiation.</td>
<td>The gathered information is presented back to the user to assist in the formulation of a prognostic judgement. Generated literature citations are offered to include with the used resources. Generated snippets by physicians may be stored for future referencing.</td>
</tr>
</table>

Outline of the PROGNOSABLE working method's steps, draft screen shot illustrations, educational program contents (learning service) and software tool characteristics (tool service).
