# Supplementary material for: Designing an evidence-based working method for medical work disability prognosis evaluation–an intervention mapping approach
Source: Front Public Health. 2023 Sep 8;11:1112683. doi: 10.3389/fpubh.2023.1112683 (PMC10516134; doi:10.3389/fpubh.2023.1112683)
Supplement: Supplementary file 6 [file Table_6.pdf]

Designing an evidence-based working method for medical disability prognosis evaluation – an intervention mapping approach

Additional file 6: Determinants, intervention functions and policy categories.

Actor	Determinants	Intervention functions	Policy categories
Physicians	Knowledge<br>Beliefs about capabilities	Education<br>Enablement<br>Training	Service provision
Clients	Beliefs about consequences	Education	Legislation
Organization	Beliefs about consequences<br>Intentions	Persuasion<br>Education<br>Incentivization<br>Environmental restructuring	Communication / marketing<br>Environmental / social planning
Professional community of physicians	Emotion	Enablement<br>Education<br>Incentivization<br>Persuasion	Communication / marketing

The table lists the best APEASE-scoring combinations from determinant – intervention function and intervention – policy category pairings [1, 2].

1. Michie S, Van Stralen MM, West R: **The behaviour change wheel: a new method for characterising and designing behaviour change interventions**. *Implementation Science* 2011, **6**(1):42.
2. Michie S, Atkins L, West R, Goosen H, van't Hof K, Mehra S: **Het gedragsveranderingswiel: 8 stappen naar succesvolle interventies**. Amsterdam: Amsterdam University Press; 2018.
