# Supplementary material for: Designing an evidence-based working method for medical work disability prognosis evaluation–an intervention mapping approach
Source: Front Public Health. 2023 Sep 8;11:1112683. doi: 10.3389/fpubh.2023.1112683 (PMC10516134; doi:10.3389/fpubh.2023.1112683)
Supplement: Supplementary file 7 [file Table_7.pdf]

Designing an evidence-based working method for medical disability prognosis evaluation – an intervention mapping approach

Additional file 7: References[i]

1. Parkes CM. Accuracy of predictions of survival in later stages of cancer. Br Med J. (1972) 2:29–31. doi: 10.1136/bmj.2.5804.29

2. Hoving JL, van der Voort R, Kok R, Verbeek JH, Hulshof CT. Het belang van een onderbouwde prognose en de rol van evidence daarbij: een survey onder verzekeringsartsen. Tijdschr Bed Verzekeringsgeneeskd. (2016) 24:465–71. doi: 10.1007/ s12498-016-0176-8

3. Christakis NA, Smith JL, Parkes CM, Lamont EB. Extent and determinants of error in doctors' prognoses in terminally ill patients: prospective cohort study. Br Med J. (2000) 320:469–73. doi: 10.1136/bmj.320.7233.469

4. Glare P, Virik K, Jones M, Hudson M, Eychmuller S, Simes J, et al. A systematic review of physicians' survival predictions in terminally ill cancer patients. Br Med J. (2003) 327:195–0. doi: 10.1136/bmj.327.7408.195

5. Mathers CD, Loncar D. Projections of global mortality and burden of disease from 2002 to 2030. PLoS Med. (2006) 3:e442. doi: 10.1371/journal.pmed.0030442

6. Anner J, Kunz R, Boer W. Reporting about disability evaluation in European countries. Disabil Rehabil. (2014) 36:848–54. doi: 10.3109/09638288.2013.821180

7. Kok R, Hoving JL, Verbeek JH, van Dijk FJ. Chapter 2.1. What knowledge questions do physicians have while performing disability evaluations and which sources do they use? In: Kok, R. (2014). Evidence-based disability evaluation. BOXPress. PhD dissertation. 's-Hertogenbosch, The Netherlands: University of Amsterdam; 2014. Persistent identifier: https://hdl.handle.net/11245/1.433673

8. LISV. Professionele herbeoordeling verzekeringsarts - Standaard. Amsterdam: Landelijk Instituut Sociale Verzekeringen (2000). p. 1–11.

9. Ankersmit JF, Chan WK, Kok R, Wind H, Hoving J. Onderbouwing van de duurzaamheid bij Wajong 2015 beoordelingen. Tijdschr Bedrijfs Verzekeringsgeneeskd. (2018) 26:518–25. doi: 10.1007/s12498-018-0316-4

10. Sackett DL, Rosenberg WM, Gray JM, Haynes RB, Richardson WS. Evidence based medicine: What it is and what it isn't. BMJ. (1996) 312:71–2. doi: 10.1136/ bmj.312.7023.71

11. Akobeng AK. Principles of evidence based medicine. Arch Dis Child. (2005) 90:837–40. doi: 10.1136/adc.2005.071761

12. Verbeek JH, van Dijk FJ, Malmivaara A, Hulshof CT, Räsänen K, Kankaanpää EE, et al. Evidence-based medicine for occupational health. Scand J Work Environ Health. (2002) 28:197–204. doi: 10.5271/sjweh.665

13. Kok R, Hoving JL, Verbeek J, Schaafsma FG, van Dijk FJ. Integrating evidence in disability evaluation by social insurance physicians. Scand J Work Environ Health. (2011) 37:494–501. doi: 10.5271/sjweh.3165

14. Kok R, Verbeek JA, Faber B, van Dijk FJ, Hoving JL. A search strategy to identify studies on the prognosis of work disability: a diagnostic test framework. BMJ Open. (2015) 5:e006315. doi: 10.1136/bmjopen-2014-006315

15. Schaafsma F, Hoving J. Prognose en het gebruik van evidence bij werkhervatting. Tijdschr Bedrijfs Verzekeringsgeneeskd. (2016) 24:487–90. doi: 10.1007/ s12498-016-0181-y

16. Hoving JL, Kok R, Ketelaar SM, Smits PB, van Dijk FJ, Verbeek JH. Improved quality and more attractive work by applying EBM in disability evaluations: a qualitative survey. BMC Med Educ. (2016) 16:77. doi: 10.1186/s12909-016-0599-z

17. Kox RJ, Hoving JL, Verbeek JH, Schouten MJ, Hulshof CT, Wind H, et al. Assessment of prognosis by physicians involved in work disability evaluation: a qualitative study. Plo S one. (2019) 14:e0212276. doi: 10.1371/journal.pone.0212276

18. Snoeck-Krygsman SP, Schaafsma FG, Donker-Cools BH, Hulshof CT, Jansen LP, Kox RJ, et al. The perceived importance of prognostic aspects considered by physicians during work disability evaluation: a survey. BMC Med Inform Decis Mak. (2022) 22:1758. doi: 10.1186/s12911-022-01758-0

19. Kok R, Hoving JL, Verbeek JH, Schaafsma FG, Smits PB, van Dijk FJ. Evaluation of a workshop on evidence-based medicine for social insurance physicians. Occup Med. (2008) 58:83–7. doi: 10.1093/occmed/kqm151

20. Verhaaf M, Hoving J. Evidence-based verzekeringsgeneeskunde. Tijdschr Bedrijfs Verzekeringsgeneeskd. (2018) 26:59–66. doi: 10.1007/s12498-018-0025-z

21. Bartholomew Eldredge LK, Markham CM, Ruiter RA, Fernández ME, Kok G, Parcel GS. Planning health promotion programs: An intervention mapping approach. New York: John Wiley and Sons (2016).

22. Michie S, Van Stralen MM, West R. The behaviour change wheel: a new method for characterising and designing behaviour change interventions. Implement Sci. (2011) 6:42. doi: 10.1186/1748-5908-6-42

23. Michie S, Atkins L, West R, Goosen H, Van’t Hof K, Mehra S. Het gedragsveranderingswiel: 8 stappen naar succesvolle interventies. Amsterdam: Amsterdam University Press (2018). 252 p.

24. Muller E, Hoving J. Visie van verzekeringsartsen op de claimbeoordeling van mensen met een beperkte levensverwachting. Tijdschr Bedrijfs Verzekeringsgeneeskd. (2018) 26:533–7. doi: 10.1007/s12498-018-0311-9

25. Barth J, de Boer WE, Busse JW, Hoving JL, Kedzia S, Couban R, et al. Inter-rater agreement in evaluation of disability: systematic review of reproducibility studies. BMJ. (2017) 356:j14. doi: 10.1136/bmj.j14

26. Anner J, Schwegler U, Kunz R, Trezzini B, de Boer W. Evaluation of work disability and the international classification of functioning, disability and health: what to expect and what not. BMC Public Health. (2012) 12:470. doi: 10.1186/1471-2458-12-470

27. Hesse B, Gebauer E, Heuft G. The IREPRO list of indicators--a tool for systematic evaluation of the prognosis for reintegration and the need for rehabilitation in the course of psychiatric disability pension assessment. Rehabilitation (Stuttg). (2007) 46:24–32. doi: 10.1055/s-2007-958527

28. van Muijen P, Duijts SF, Kornet-van der Aa DA, van der Beek AJ, Anema JR. Work disability assessment of cancer survivors: insurance physicians' perspectives. Occup Med (Lond). (2015) 65:558–63. doi: 10.1093/occmed/kqv098

29. Louwerse I, Huysmans M, van Rijssen H, Gielen C, van der Beek A, Anema J. Use of a decision support tool on prognosis of work ability in work disability assessments: an experimental study among insurance physicians. J Occup Rehabil. (2020) 31:185–96. doi: 10.1007/s10926-020-09907-w

30. Donker-Cools B, Wind H, Frings-Dresen MHW. Acquired brain injury and return to work: the feasibility of a training program for insurance physicians. Disabil Rehabil. (2019) 42:1480–6. doi: 10.1080/09638288.2018.1527400

31. Sallis JF, Cervero RB, Ascher W, Henderson KA, Kraft MK, Kerr J. An ecological approach to creating active living communities. Annu Rev Public Health. (2006) 27:297–322. doi: 10.1146/annurev.publhealth.27.021405.102100

32. Green LW, Kreuter MW. The Precede-Proceed model of Health Program Planning and Evaluation. (2020) Available at: http://www.lgreen.net/precede.htm.

33. Porter CM. Revisiting Precede–Proceed: a leading model for ecological and ethical health promotion. Health Educ J. (2016) 75:753–64. doi: 10.1177/0017896915619645

34. Fassier JB, Sarnin P, Rouat S, Peron J, Kok G, Letrilliart L, et al. Interventions developed with the intervention mapping protocol in work disability prevention: a systematic review of the literature. J Occup Rehabil. (2019) 29:11–24. doi: 10.1007/ s10926-018-9776-8

35. van Oostrom SH, Anema JR, Terluin B, Venema A, de Vet HC, van Mechelen W. Development of a workplace intervention for sick-listed employees with stress-related mental disorders: intervention mapping as a useful tool. BMC Health Serv Res. (2007) 7:127. doi: 10.1186/1472-6963-7-127

36. Vermeulen SJ, Anema JR, Schellart AJ, van Mechelen W, van der Beek AJ. Intervention mapping for development of a participatory return-to-work intervention for temporary agency workers and unemployed workers sick-listed due to musculoskeletal disorders. BMC Public Health. (2009) 9:216. doi: 10.1186/1471- 2458-9-216

37. Zwerver F, Schellart AJ, Anema JR, Rammeloo KC, van der Beek AJ. Intervention mapping for the development of a strategy to implement the insurance medicine guidelines for depression. BMC Public Health. (2011) 11:9. doi: 10.1186/1471-2458-11-9

38. Michie S, Johnston M, Abraham C, Lawton R, Parker D, Walker A, et al. Making psychological theory useful for implementing evidence based practice: a consensus approach. Qual Saf Health Care. (2005) 14:26–33. doi: 10.1136/qshc.2004.011155

39. Cane J, O'Connor D, Michie S. Validation of the theoretical domains framework for use in behaviour change and implementation research. Implement Sci. (2012) 7:37. doi: 10.1186/1748-5908-7-37

40. Fishbein M, Triandis HC, Kanfer FH, Becker M, Middlestadt SE, Eichler A. Factors influencing behavior and behavior change. Handb Health Psychol. (2001) 1:3–17.

41. Jaccard J, Dodge T, Dittus P. Parent-adolescent communication about sex and birth control: a conceptual framework. New Dir Child Adolesc Dev. (2002) 2002:9–42. doi: 10.1002/cd.48

42. Montaño D, Kaspryzk D. Theory of reasoned action, theory of planned behavior, and the integrated behavior model In: K Glanz, BK Rimer and K Viswanath, editors. Health behavior and health education: theory, research, and practice. Hoboken, NJ: Jossey-Bass (2008). 67–92.

43. Louwerse I, Huysmans MA, van Rijssen JHJ, Overvliet J, van der Beek AJ, Anema JR. Preferences regarding the way of use and design of a work ability prognosis support tool: a focus group study among professionals. Disabil Rehabil. (2019) 43:2031–7. doi: 10.1080/09638288.2019.1693643

44. De Boer W. Quality of evaluation of work disability. Academisch proefschrift Universiteit van Amsterdam Hoofddorp (2010).

45. Michie S, Carey RN, Johnston M, Rothman AJ, de Bruin M, Kelly MP, et al. From theory-inspired to theory-based interventions: a protocol for developing and testing a methodology for linking behaviour change techniques to theoretical mechanisms of action. Ann Behav Med. (2018) 52:501–12. doi: 10.1007/s12160-016-9816-6

46. Michie S, Richardson M, Johnston M, Abraham C, Francis J, Hardeman W, et al. The behavior change technique taxonomy (v1) of 93 hierarchically clustered techniques: building an international consensus for the reporting of behavior change interventions. Ann Behav Med. (2013) 46:81–95. doi: 10.1007/s12160-013-9486-6

47. Michie S, West R, Campbell R, Brown J, Gainforth H. ABC of behaviour change theories: An essential resource for researchers. Policy Makers and Practitioners: Silverback Publishing (2014). 501 p.

48. Raad voor Gezondheidsonderzoek (RGO). Advies onderzoek verzekeringsgeneeskunde. The Hague: Raad voor Gezondheidsonderzoek (RGO) June 2004. Report No.: Publication No. 44 Contract No.: 44. (2004)

49. Van Dijk FJ, Verbeek JH, Hoving JL, Hulshof CT. A knowledge infrastructure for occupational safety and health. J Occup Environ Med. (2010) 52:1262–8. doi: 10.1097/ JOM.0b013e318202f2c5

50. Slebus FG, Sluiter JK, Kuijer PPF, Willems JH, Frings-Dresen MH. Work-ability evaluation: a piece of cake or a hard nut to crack? Disabil Rehabil. (2007) 29:1295–300. doi: 10.1080/09638280600976111

51. Anner J, Brage S, Donceel P, Falez F, Freudenstein R, Oancea C, et al. Validation of the EUMASS Core set for medical evaluation of work disability. Disabil Rehabil. (2013) 35:2147–56. doi: 10.3109/09638288.2013.771709

52. World Health Organization. International classification of functioning, disability and health: ICF. Geneva: World Health Organization (2001).

53. De Wit M, Wind H, Hulshof CT, Frings-Dresen MH. Person-related factors associated with work participation in employees with health problems: a systematic review. Int Arch Occup Environ Health. (2018) 91:497–512. doi: 10.1007/ s00420-018-1308-5

54. Brage S, Donceel P, Falez F. Development of ICF core set for disability evaluation in social security. Disabil Rehabil. (2008) 30:1392–6. doi: 10.1080/09638280701642950

55. Finger ME, Escorpizo R, Glässel A, Gmünder HP, Lückenkemper M, Chan C, et al. ICF Core set for vocational rehabilitation: results of an international consensus conference. Disabil Rehabil. (2012) 34:429–38. doi: 10.3109/09638288.2011.608145

56. Sengers JH, Abma FI, Wilming L, Roelofs P, Heerkens YF, Brouwer S. Content validation of a practice-based work capacity assessment instrument using ICF Core sets. J Occup Rehabil. (2020) 31:293–315. doi: 10.1007/s10926-020-09918-7

57. Borland R. CEOS theory: a comprehensive approach to understanding hard to maintain behaviour change. Appl Psychol Health Well Being. (2017) 9:3–35. doi: 10.1111/ aphw.12083

58. Kunz R, Verbel A, Weida-Cuignet R, Hoving JL, Weinbrenner S, Friberg E, et al. Evidence needs, training demands, and opportunities for knowledge translation in social security and insurance medicine: a European survey. J Rehabil Med. (2021) 53:jrm00179. doi: 10.2340/16501977-2821

59. Maibach EW, Cotton D. Moving people to behavior change: A staged social cognitive approach to message design In: E Maibach and RL Parrott, editors. Designing health messages: approaches from communication theory and public health practice. Thousand Oaks, CA: Sage Publications, Inc. (1995). 41–64.

60. Greidanus MA, de Boer AGEM, Tiedtke CM, Frings-Dresen MHW, de Rijk AE, Tamminga SJ. Supporting employers to enhance the return to work of cancer survivors: development of a web-based intervention (MiLES intervention). J Cancer Surviv. (2020) 14:200–10. doi: 10.1007/s11764-019-00844-z

61. Fassier JB, Lamort-Bouche M, Broc G, Guittard L, Peron J, Rouat S, et al. Developing a return to work intervention for breast Cancer survivors with the intervention mapping protocol: challenges and opportunities of the needs assessment. Front Public Health. (2018) 6:35. doi: 10.3389/fpubh.2018.00035

62. Vooijs M, Bossen D, Hoving JL, Wind H, Frings-Dresen MH. A training programme facilitating guideline use of occupational health professionals: a feasibility study. BMC Med Educ. (2018) 18:226. doi: 10.1186/s12909-018-1223-1

63. Los FS, van der Molen HF, Hulshof CTJ, de Boer A. Supporting occupational physicians in the implementation of Workers' health surveillance: development of an intervention using the behavior change wheel framework. Int J Environ Res Public Health. (2021) 18:1939. doi: 10.3390/ijerph18041939

64. Louwerse I, Huysmans MA, van Rijssen JH, Schaafsma FG, Weerdesteijn KH, van der Beek AJ, et al. Predicting future changes in the work ability of individuals receiving a work disability benefit: weighted analysis of longitudinal data. Scand J Work Environ Health. (2019) 46:168–76. doi: 10.5271/sjweh.3834

65. De Wit M, Horreh B, Daams JG, Hulshof CT, Wind H, de Boer AG. Interventions on cognitions and perceptions that influence work participation of employees with chronic health problems: a scoping review. BMC Public Health. (2020) 20:1–17. doi: 10.1186/s12889-020-09621-5

66. Hesse B. Entwicklung einer Indikatorenliste zur sozialmedizinischen Beurtei-lung der Reintegrationsprognose und der Rehabilitationsbedürftigkeit bei Rentenantragstellern mit psychischen Erkrankungen (IREPRO). des NRW-Forschungsverbundes Rehabilitationswissenschaften. pp. 134–146. (2006).

67. de Leeuw R, Scheele F, Walsh K, Westerman M. A 9-step theory-and evidencebased postgraduate medical digital education development model: empirical development and validation. JMIR Med Educ. (2019) 5:e13004. doi: 10.2196/13004

68. Coomarasamy A, Khan KS. What is the evidence that postgraduate teaching in evidence based medicine changes anything? A systematic review. BMJ. (2004) 329:1017. doi: 10.1136/bmj.329.7473.1017

69. van Dijk F, Caraballo-Arias Y. Where to find evidence-based information on occupational safety and health? Ann Glob Health. (2021) 87:6. doi: 10.5334/ aogh.3131

70. Michie S, Johnston M, Francis J, Hardeman W, Eccles M. From theory to intervention: mapping theoretically derived behavioural determinants to behaviour change techniques. Appl Psychol. (2008) 57:660–80. doi: 10.1111/j.1464-0597.2008.00341.x

71. Colquhoun HL, Squires JE, Kolehmainen N, Fraser C, Grimshaw JM. Methods for designing interventions to change healthcare professionals' behaviour: a systematic review. Implement Sci. (2017) 12:30. doi: 10.1186/s13012-017-0560-5

[i] Additional files 1 to 4 and 6 contain separate reference lists.
