# Supplementary material for: Designing an evidence-based working method for medical work disability prognosis evaluation–an intervention mapping approach
Source: Front Public Health. 2023 Sep 8;11:1112683. doi: 10.3389/fpubh.2023.1112683 (PMC10516134; doi:10.3389/fpubh.2023.1112683)
Supplement: Supplementary file 8 [file Table_8.pdf]

# Designing an evidence-based working method for medical disability prognosis evaluation – an intervention mapping approach

## Additional file 8: Supplementary material

Additional file 1 – Intervention Mapping steps
*Additional file 1: The six steps of Intervention Mapping*

Additional file 2 – TDF COM-B
*Additional file 2: Domains of the Theoretical Domains Framework (TDF) and their contribution to the components of the COM-B model (Capacity-Opportunity-Motivation⟶Behavior).*

Additional file 3 – Study flow
*Additional file 3: Study flow from determinants to theory selection*

Additional file 4 – Needs and contexts
*Additional file 4: Needs and contexts of the four actors: physicians, clients, the organization and the professional community of the physicians.*

Additional file 5 – Prognosable
*Additional file 5: The intervention Prognosable, consisting of a working method, educational service and tool service.*

The outline shows the Prognosable working method's steps, draft screen shot illustrations, educational program contents (learning service) and software tool characteristics (tool service).

Additional file 6- APEASE
*Additional file 6: Selected determinants, intervention functions and policy categories.*

The table lists the best APEASE-scoring combinations from determinant – intervention function and intervention – policy category pairings.

Additional file 7 – References
*Additional file 7: References used in manuscript and supplements*

Additional file 8 – Supplementary material
*Additional file 8: Overview of supplementary tables and figures*
